# Network Model of Immune Responses Reveals Key Effectors to Single and Co-infection Dynamics by a Respiratory Bacterium and a Gastrointestinal Helminth

**DOI:** 10.1371/journal.pcbi.1002345

**Published:** 2012-01-12

**Authors:** Juilee Thakar, Ashutosh K. Pathak, Lisa Murphy, Réka Albert, Isabella M. Cattadori

**Affiliations:** 1 Center for Infectious Disease Dynamics, The Pennsylvania State University, University Park, Pennsylvania, United States of America; 2 Department of Pathology, Yale University School of Medicine, New Haven, Connecticut, United States of America; 3 Department of Biology, The Pennsylvania State University, University Park, Pennsylvania, United States of America; 4 Division of Animal Production and Public Health, Veterinary School, University of Glasgow, Glasgow, United Kingdom; 5 Department of Physics, The Pennsylvania State University, University Park, Pennsylvania, United States of America; Utrecht University, Netherlands

## Abstract

Co-infections alter the host immune response but how the systemic and local processes at the site of infection interact is still unclear. The majority of studies on co-infections concentrate on one of the infecting species, an immune function or group of cells and often focus on the initial phase of the infection. Here, we used a combination of experiments and mathematical modelling to investigate the network of immune responses against single and co-infections with the respiratory bacterium *Bordetella bronchiseptica* and the gastrointestinal helminth *Trichostrongylus retortaeformis*. Our goal was to identify representative mediators and functions that could capture the essence of the host immune response as a whole, and to assess how their relative contribution dynamically changed over time and between single and co-infected individuals. Network-based discrete dynamic models of single infections were built using current knowledge of bacterial and helminth immunology; the two single infection models were combined into a co-infection model that was then verified by our empirical findings. Simulations showed that a T helper cell mediated antibody and neutrophil response led to phagocytosis and clearance of *B. bronchiseptica* from the lungs. This was consistent in single and co-infection with no significant delay induced by the helminth. In contrast, *T. retortaeformis* intensity decreased faster when co-infected with the bacterium. Simulations suggested that the robust recruitment of neutrophils in the co-infection, added to the activation of IgG and eosinophil driven reduction of larvae, which also played an important role in single infection, contributed to this fast clearance. Perturbation analysis of the models, through the knockout of individual nodes (immune cells), identified the cells critical to parasite persistence and clearance both in single and co-infections. Our integrated approach captured the within-host immuno-dynamics of bacteria-helminth infection and identified key components that can be crucial for explaining individual variability between single and co-infections in natural populations.

## Author Summary

Infections with different infecting agents can alter the immune response against any one parasite and the relative abundance and persistence of the infections within the host. This is because the immune system is not compartmentalized but acts as a whole to allow the host to maintain control of the infections as well as repair damaged tissues and avoid immuno-pathology. There is no comprehensive understanding of the immune responses during co-infections and of how systemic and local mechanisms interact. Here we integrated experimental data with mathematical modelling to describe the network of immune responses of single and co-infection by a respiratory bacterium and a gastrointestinal helminth. We were able to identify key cells and functions responsible for clearing or reducing both parasites and showed that some mechanisms differed between type of infection as a result of different signal outputs and cells contributing to the immune processes. This study highlights the importance of understanding the immuno-dynamics of co-infection as a host response, how immune mechanisms differ from single infections and how they may alter parasite persistence, impact and abundance.

## Introduction

Hosts that are immunologically challenged by one infection often show increased susceptibility to a second infectious agent, whether a micro- or a macro-parasite. Changes in the immune status and polarization of the response towards one parasite can indeed facilitate the establishment and survival of a second parasitic species [1]–[3]. At the level of the individual host, this can be described as an immune system that has to optimize the specificity and effectiveness of the responses against different infections while engaging in secondary but equally important functions, like tissue repair or avoiding immuno-pathology. Systemic cross-regulatory processes and bystander effects by T helper cells (Th) maintain control of these functions both at the systemic and local level [4]–[8]. Concurrent parasite infections are regulated by and affect these mechanisms [2], [4], [9]–[14]. They can also influence each other directly, when sharing the same tissue [15]–[16] or through the immune system via passive effects or active manipulation of the immune components, if colonizing different organs [4], [9]–[14].

Empirical work on bacteria-macroparasite co-infections has often found that the development of a Th2 mediated response towards the helminth leads to a reduction of the protective Th1 cytokine response against the bacteria and a more severe bacteria-induced pathology [4], [11]–[14], although a decrease of tissue atrophy has also been observed [17]–[18]. The suppression of Th1 cell proliferation acts both on the inductors and effectors and is mainly driven by the repression of the IFNγ mediated inflammatory activity during the early stages of the infection. However, the degree of the T helper cell polarization and the kinetics of effectors depend on the type, intensity and duration of the co-infection, over and above the very initial immune status of the host. Since host immunity is both a major selective pressure for parasite transmission and host susceptibility to re-infections, the presence of one infection can have major consequences for the spread and persistence of the second infection. For example, *Mycobacterium tuberculosis* induces more severe disease when concurrent with intestinal helminths, suggesting increased host infectiousness and bacterial transmission compared to single infected individuals [14].

Understanding how the infection by a second parasite species can influence the network of immune processes and the polarization towards one of the infecting agents requires the quantification of the immune components both at the systemic level and at the local site of infection, and the ability to follow the kinetics of these processes over time. The immunology of co-infection often considers the Th1/Th2 paradigm a tractable simplification of the overall immune response and its main functions. Yet, this approach tells us only half of the story, namely the systemic component. Indeed, organ compartmentalization and tissue specificity create well defined host-parasite environments that contribute to, as well as are modulated by, the immune system as a whole [19]–[20]. This brings us to the questions: what are the key processes and components that capture the essence of immune mediated parasite interactions in co-infections? And, how do these differ from single infections?

To address these questions we used a combination of laboratory experiments and network-based discrete dynamic modelling, and examined changes in the immune response against single and co-infection with the respiratory bacterium *Bordetella bronchiseptica* and the gastrointestinal helminth *Trichostrongylus retortaeformis*, two common infections of the European rabbit (*Oryctolagus cuniculus*). Both parasites cause persistent infections that occur with high prevalence and intensity in free-living rabbit populations [21]–[22]. *B. bronchiseptica* is a gram-negative bacterium that colonizes the respiratory tract through oral-nasal transmission and usually results in asymptomatic infections. *B. bronchiseptica* has been largely isolated in wildlife, pets and livestock but rarely in humans [23] where it is out-competed by the human-specific *Bordetella pertussis* and *Bordetella parapertussis*, the etiological agents of whooping cough [24]. Previous empirical and modelling work in a murine system showed that the bacterium induces anti-inflammatory responses by modulating Th regulation, thereby facilitating bacterial establishment and proliferation [8], [25]. However, hosts successfully counteract the pathogen mediated inhibitions by activating a protective Th1 cell mediated IFNγ response, which leads to bacterial clearance from the lower respiratory tract, but not the nasal cavity, via Fc receptor mediated phagocytosis [25]–[27]. Our recent laboratory studies of rabbits infected with *B. bronchiseptica* agree with the general findings of bacterial clearance from the lower respiratory tract but persistence in the nasal cavity [28].

The gastrointestinal helminth *T. retortaeformis* has a direct life cycle and colonizes the small intestine following ingestion of pasture contaminated with infective third stage larvae (L3). The majority of larvae settle in the duodenum where they develop into adults in about 11 days [29]. A model of the seasonal dynamics of the *T. retortaeformis*-rabbit interaction suggested that acquired immunity develops proportionally to the accumulated exposure to infection and successfully reduces helminth intensity in older hosts [21], [30]. These results were recently confirmed by challenging laboratory rabbits with a primary infection of *T. retortaeformis* where the quick production of antibodies and eosinophils was associated with the consistent reduction but not complete clearance of the helminth by 120 days post challenge [31].

Based on previous studies on bacteria-macroparasite co-infections and our recent work on the rabbit system, we hypothesized that during a *B. bronchiseptica*-*T. retortaeformis* co-infection the presence of helminths will delay bacterial clearance from the respiratory tract but there will be no change in helminth abundance in the small intestine. We predicted a *T. retortaeformis* mediated Th2 polarization at the systemic level and a bystander effect in the distal respiratory tract. This will have suppressed IFNγ, resulting in the enhancement of bacterial intensity and deferred clearance in the lower respiratory tract compared to single infection. We also expected the Th2 systemic environment to control helminth abundance but not to change the numbers compared to the single infection. To examine our hypothesis, laboratory data on single infections were used to build discrete dynamic models describing the immune processes generated in response to each infection. The two single infection models were then connected through the cross-modulation of Th cells and the cytokine network at the systemic level, and allowed to reflect changes in these interactions at the local level. The resulting co-infection model and the dynamics of the parasites were finally compared with our laboratory experiment of bacteria-helminth co-infection to confirm the correctness of the model. Lastly, we examined the robustness of the immune networks with respect to the deactivation of single immune nodes by simulated knockout laboratory experiments. In other words, we tested the role of a large number of immune components, how their knockout affected the dynamics of infection and how the system converged into a potentially novel stable state. This allowed us to elucidate the immune key mechanisms and pathways behind the observed dynamics and the relative differences between single and co-infection.

## Results

**Figure 1:**
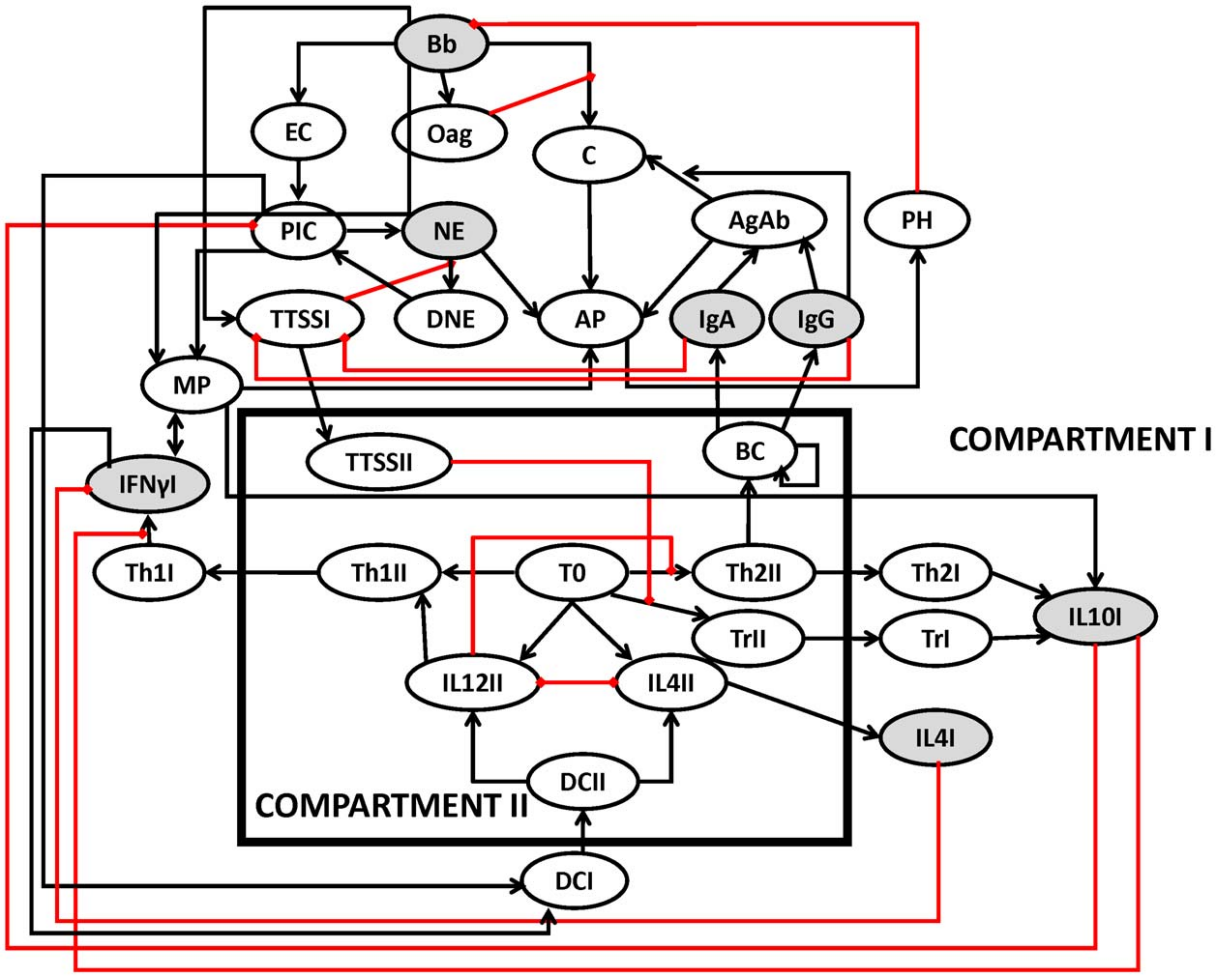
Network of immune components considered in single *B. bronchiseptica* infection. Ovals represent network nodes and indicate the node name in an abbreviated manner. Compartment I denotes the nodes in the lungs and Compartment II combines the nodes at systemic level. Terminating black arrows on an edge indicate positive effects (activation) and terminating red blunt segments indicate negative effects (inhibition). Grey nodes have been quantified in the single laboratory experiment. Abbreviations: **Bb**: *B. bronchiseptica*; **Oag**: O-antigen; **IL4II**: Interleukin 4 in the systemic compartment; **NE**: Recruited neutrophils; **IL12I**: Interleukin 12 in lungs; **IgA**: Antibody A; **C**: Complement; **TrII**: T regulatory cells in the systemic compartment; **IL4I**: Interleukin 4 in the lungs; **Th2II**: Th2 cells in the systemic compartment; **TrI**: T regulatory cells in the lungs; **Th2I**: Th2 cells in the lungs; **IL10II**: Interleukin 10 in the lymph nodes; **TTSSII**: Type three secretion system in the lymph nodes; **TTSSI**: Type three secretion system in the lungs; **IgG**: Antibody G; **IL10I**: Interleukin 10 in the lungs; **IFNγI**: Interferon gamma in the lungs; **IL12II**: Interleukin 12 in the systemic compartment; **BC**: B cells; **DCII**: Dendritic cells in the systemic compartment; **DCI**: Dendritic cells in the lungs; **Th1I**: T helper cell subtype I in the lungs; **PIC**: Pro-inflammatory cytokines; **Th1II**: T helper cell subtype I in the systemic compartment **EC**: Epithelial cells; **AP**: Activated phagocytes; **T0**: Naïve T cells; **AgAb**: Antigen-antibody complexes; **MP**: Macrophages in the lungs; **DNE**: dead neutrophils; **PH**: Phagocytosis.

**Figure 2:**
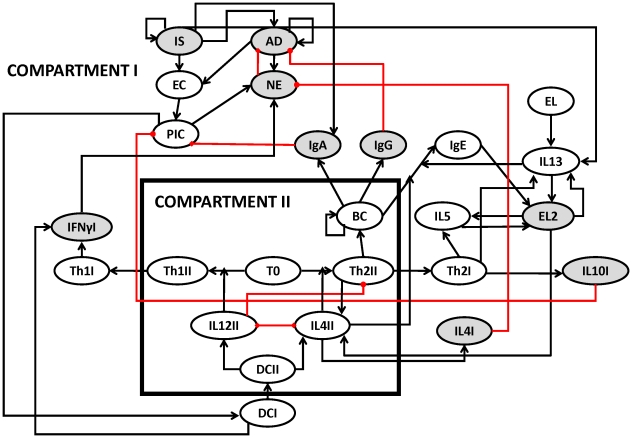
Network of immune components considered in single *T. retortaeformis* infection. Grey nodes have been quantified in the single laboratory experiment. Abbreviations: **IS**: Larvae; **AD**: Adult; **IL4II**: Interleukin 4 in the systemic compartment; **NE**: Recruited neutrophils; **IgA**: Antibody A; **IL4I**: Interleukin 4 in the small intestine; **Th2II**: Th2 cells in the systemic compartment; **Th2I**: Th2 cells in the small intestine; **IgG**: Antibody G; **IgE**: Antibody E; **IL10I**: Interleukin 10 in the small intestine; **IFNγI**: Interferon gamma in the small intestine; **IL12II**: Interleukin 12 in the systemic compartment; **BC**: B cells; **DCII**: Dendritic cells in the systemic compartment; **DCI**: Dendritic cells in the small intestine; **Th1I**: T helper cells subtype I in the small intestine; **PIC**: Pro-inflammatory cytokines; **Th1II**: T helper cells subtype I in systemic compartment **EC**: Epithelial cells the small intestine; **T0**: Naïve T cells; **EL2**: recruited eosinophils; **EL**: resident eosinophils; **IL13**: Interleukin 13; **IL5**: Interleukin 5. Additional details in Figure 1.

The causal interactions between the immune components activated by *B. bronchiseptica* and *T. retortaeformis* were assembled in the form of two distinct pathogen-specific networks of immune responses. The network of interactions against *B. bronchiseptica* was based on the infection in the lungs, the crucial organ for bacterial clearance, and constructed following Thakar et al. [8] and the current knowledge of the dynamics of *B. bronchiseptica* infection in mice (**Fig. 1**). There is a rich literature on the immunology of gastrointestinal helminth infections and important general features can be identified despite the fact that these mechanisms are often species-specific. The immune network against *T. retortaeformis* was built on the knowledge of helminth infections in mice [6]–[7] and focused on the duodenum (the first section of the small intestine), where the majority of *T. retortaeformis* colonization and immune activity was observed (**Fig. 2**) [29], [31]. Both networks were characterized by two connected compartments: Compartment I represented the immune interactions at the local site of infection, the lungs or duodenum, while Compartment II described the systemic site of T and B cell activation and differentiation, for example, the lymph node.

The networks were then developed into discrete dynamic models [32]. Discrete dynamic modelling has been proven to be a feasible and useful approach to qualitatively characterize systems where the detailed information necessary for quantitative models is lacking [32]–[33]. For our purpose to examine the pattern of immune responses to single and co-infection at the local and systemic level and, importantly, to highlight key interactions and cells that generated the pattern observed, the framework of the discrete dynamic Boolean model appeared to be a robust and tractable choice [34]–[36], given that the kinetics and timescales of many of the immune interactions is unknown in the rabbit system. Each node (e.g. immune cell) was categorized by two qualitative states, ON and OFF, which are determined from the regulation of the focal node by upstream nodes given in the network. This regulation is given by a Boolean transfer function [32], [34]–[35] (see Materials and Methods, and Supplement Text S1). The nodes in the ON state are assumed to be above an implicit threshold that can be defined as the concentration necessary to activate downstream immune processes; below this threshold the node is in an OFF state. To follow the dynamical status of the system through time, we repeatedly applied the Boolean transfer functions for each node until a steady state (i.e. clearance of the pathogen) was found. To determine the node consensus activity over time (i.e. the time course of cell concentration or parasite numbers shared by multiple trajectories) we ran the simulations 100 times by randomly sampling timescales and plotted each node activity profile, defined as the proportion of simulations in which the node is in the ON state as a function of time (additional details in the Materials and Methods) [37]–[38]. This procedure is similar to characterizing the consensus behaviour of a population of infected hosts that exhibit individual-to-individual variation.

To construct the single infection models, we formulated the Boolean transfer functions from the current knowledge of the immune regulatory processes and in case of ambiguity we iteratively modified the transfer function by comparing the simulated dynamic output with our empirical results on single infection and with immune knockout studies (a detailed example is reported in the Materials and Methods). Finally, to examine the relative importance of the immune components, we perturbed each node by setting their status to OFF and monitored parasite activity up to the time-step required for parasite clearance/reduction in the unperturbed system. Any increase in the infection activity following the knockout of an immune node -which may cascade to the connected downstream nodes- indicated the importance of this node for parasite clearance. Nodes whose deactivation led to long term persistence, represented by parasite activity equal to 1, were classified as essential for clearance. This procedure allowed us to mimic laboratory experiments of single immune component knockouts and to follow the consequences on parasite clearance.

### *B. bronchiseptica* single infection

**Figure 3:**
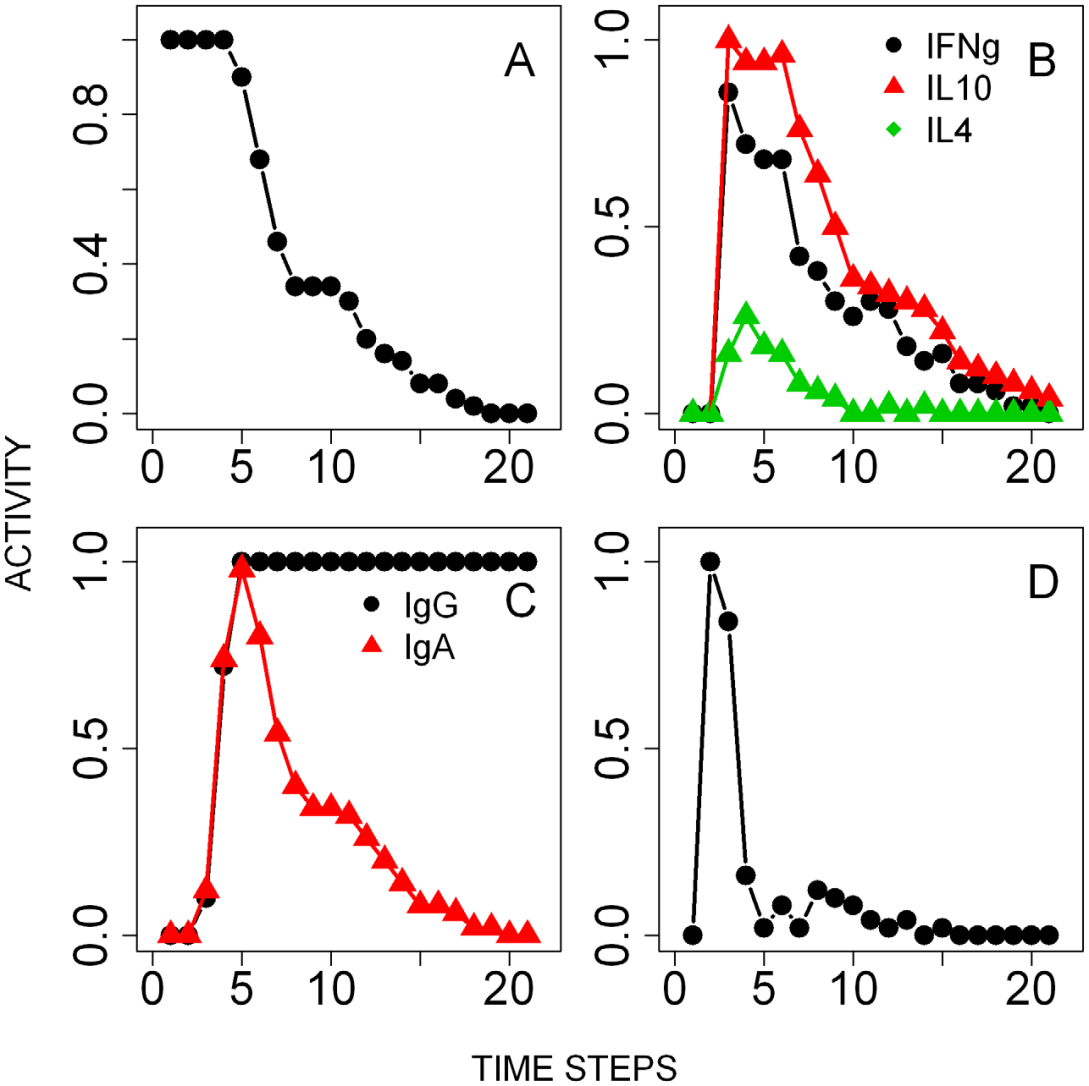
Results of the simulations of the time course of the single *B. bronchiseptica* infection. Activity profiles (the probability of the node being in an ON state at a given time-step) are reported for: **A**- Bacterial colonies in the lungs. **B**- Cytokines, IFNγ, IL4 and IL10, in the lungs. **C**- Serum antibodies. **D**- Peripheral neutrophils.

The onset of *B. bronchiseptica* infection in the lungs was simulated by setting the state of the bacteria node ON and the state of the nodes of the immune response OFF (**Fig. 3A**). As the infection proceeded, and consistent with our empirical work [28], IFNγ and IL10 expression rapidly peaked and then slowly decreased below the threshold through the course of the infection (**Fig. 3B**). *B. bronchiseptica* has been suggested to induce IL10 production by T cell subtypes, which inhibits IFNγ in the lower respiratory tract [25]. By explicitly including the bacteria mediated up-regulation of IL10, through the type III secretion system (TTSS) modulation of T regulatory cells (Treg), we were able to capture the establishment of the bacteria in the lungs followed by their immune-mediated reduction and clearance. Activation of B cells by T helper cells led to the prompt increase of peripheral antibodies (serum IgG and IgA), in line with empirical data [26]–[27], [39]. Serum IgG reached and maintained long-lasting above-threshold saturation in all simulations whereas IgA activity dropped along with *B. bronchiseptica* and was turned off after 15 time-steps (see Materials and Methods) (**Fig. 3C**). The rapid recruitment of peripheral neutrophils to the lungs was possible through pro-inflammatory cytokine mediated signalling (**Fig. 3D**), while macrophages were recruited by IFNγ secreted by Th1 cells. The activation of neutrophils and macrophages by antibodies, via the antibody-antigen complex and complement nodes (see **Fig. 1**), led to bacterial phagocytosis and clearance from the lungs within 20 time steps, in agreement with our empirical work.

**Figure 4:**
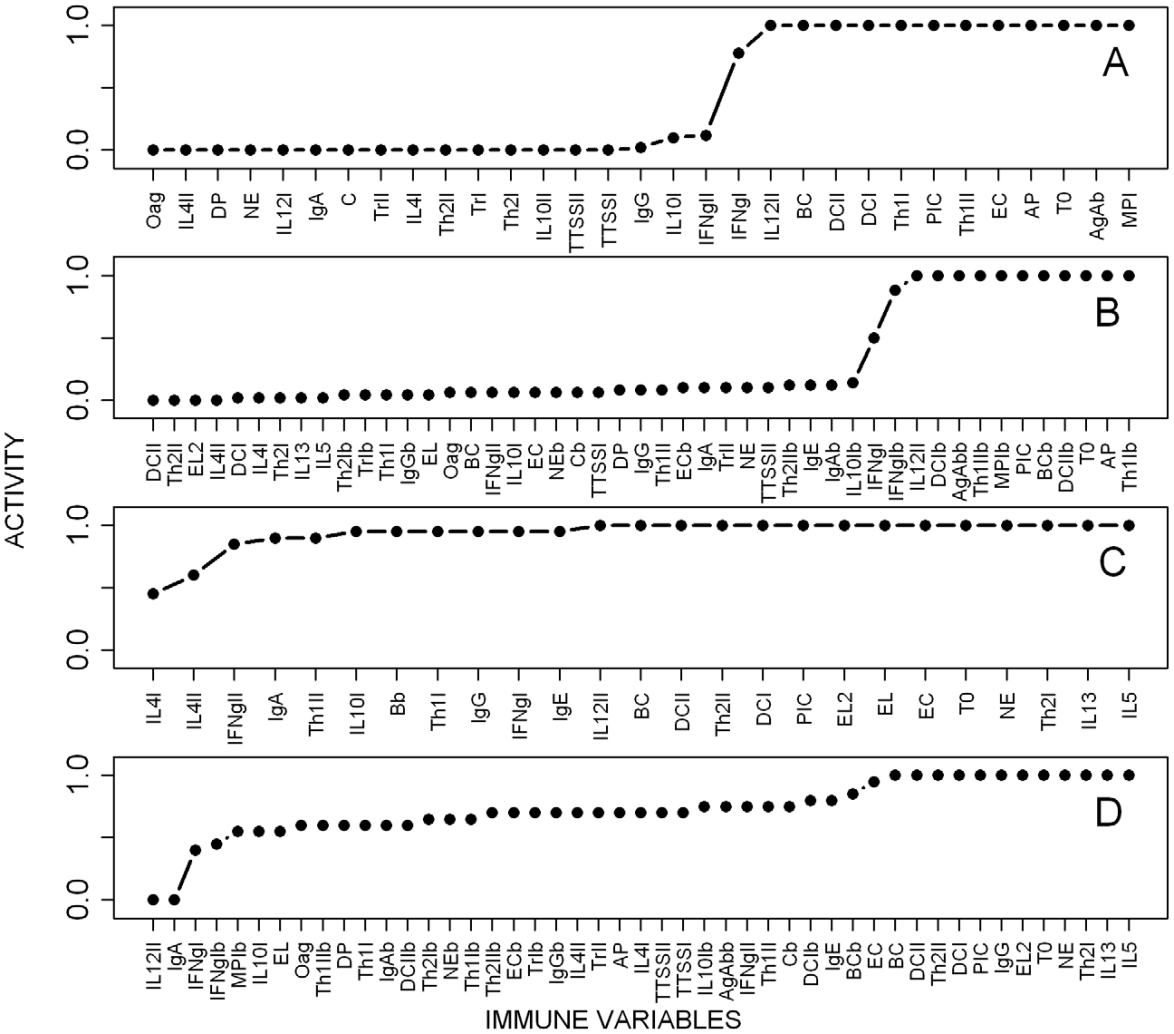
Parasite activity at the 20^th^ time step from simulations where network nodes were individually knocked out (from 100 replicates). **A**- *B. bronchiseptica* in single infection. **B**- *B. bronchiseptica* in co-infection. **C**- *T. retortaeformis* in single infection. **D**- *T. retortaeformis* in co-infection. Explanation of the abbreviations is reported in Figure 1, Figure 2 and Text S1.

The relative importance of the different immune components was then explored by knocking off single nodes and monitoring the level of bacterial intensity at the 20^th^ time-step, the time required for *B. bronchiseptica* clearance from the lungs in the unperturbed system. The perturbation results reproduced the observations from *B. bronchiseptica* infections in the respective empirical knockout experiments (**Fig. 4A**) [8]. For example, it has been observed that *B. bronchiseptica* can persist in large numbers in mice where T0, Th1 or B cells are depleted [8]; the key role of these nodes was confirmed by our model. The simulations also highlighted the crucial role of pro-inflammatory responses, dendritic cells, macrophages and IL12 as their inactive state resulted in bacterial persistence (**Fig. 4A**). In contrast, knocking out IL4 or any of the 15 remaining nodes of the network did not increase the activity of the node *Bordetella*.

### *T. retortaeformis* single infection

**Figure 5:**
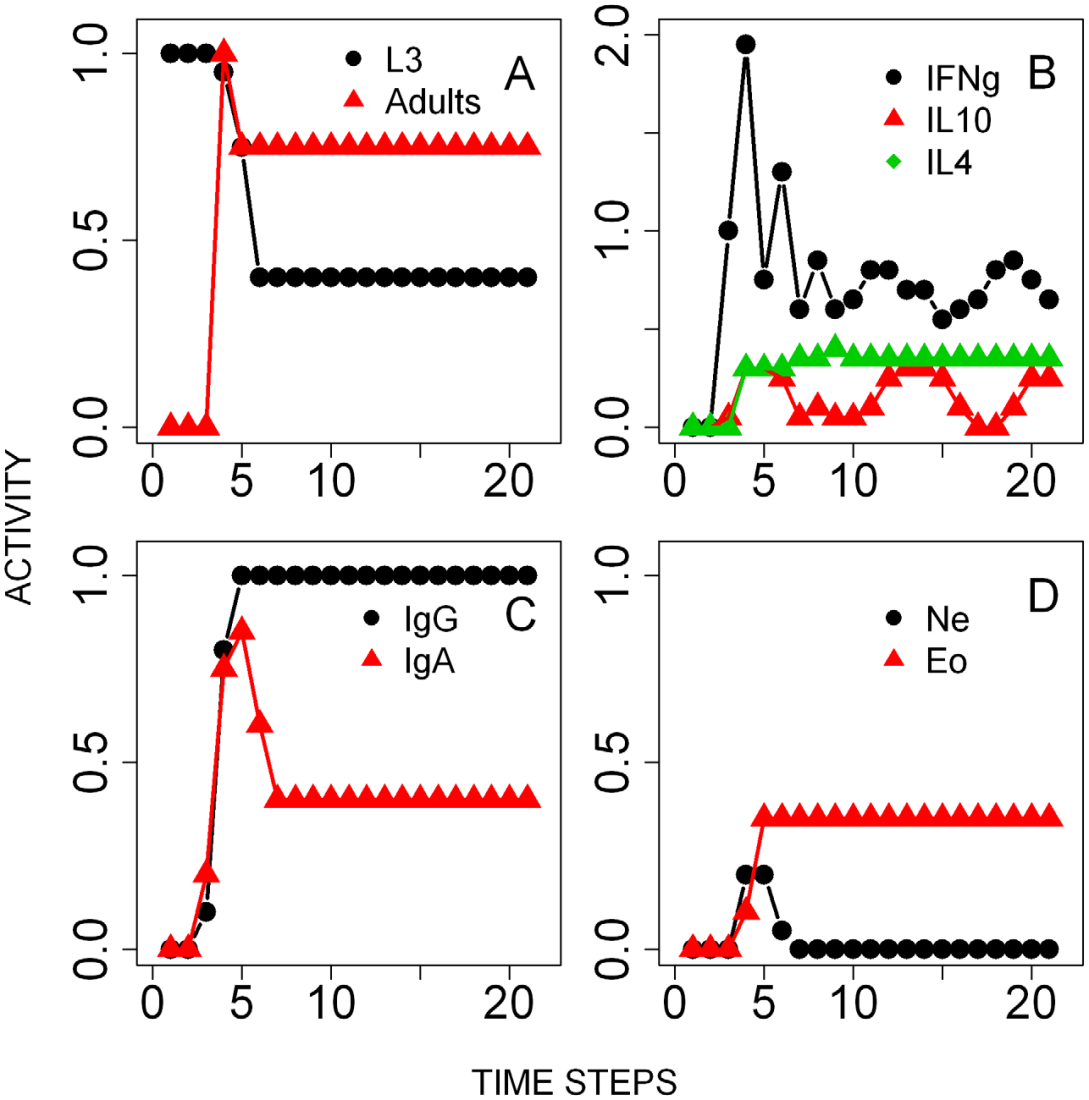
Results of the simulations of the time course of the single *T. retortaeformis* infection. Activity profiles (the probability of the node being in an ON state at a given time-step) are reported for: **A**- Third stage infective larvae (L3) and adults. **B**- Cytokines, IFNγ, IL4 and IL10 in the duodenum. **C**- Mucus antibodies against helminth adult parasites. **D**- Peripheral eosinophils and neutrophils. Note that the IFNγ concentration range is between 0–2 to describe additional non-immune mediated activation of that node by the tissue damage (details in the Results).

The infection of *T. retortaeformis* was simulated by setting the state of the infective larvae node ON and the immune nodes OFF (**Fig. 5A**). Ingested larvae were either killed by eosinophils, in a probabilistic manner [40]–[41], or successfully developed into adults. Adults started to appear after 2 time-steps, mimicking the natural development of infective third stage larvae into adults. Following the infection, IFNγ rapidly peaked after two time steps while IL4 and IL10 activation followed with a delay, in line with empirical findings (**Fig. 5B**) [31]. The initial vigorous expression of IFNγ was driven by dendritic cells, probably as an inflammatory response to the infiltration of microflora and bacteria into the damaged mucosa during the establishment of larvae [31]. This was modelled by turning the activity of the local IFNγ ON if sufficiently stimulated by dendritic cells; the subsequent IFNγ activation occurred through a Th1 cell response. For the interpretation of Fig. 5B, the fraction of IFNγ activity that occurred from 0 to 1 was due to a Th1 response while above 1 was caused by dendritic cells. Dendritic cells also activated the Th2 cell mediated expression of IL4 and as this arm of the immune response developed, IFNγ decreased although remained in an active state throughout the infection (**Fig. 5B**). IL10 expression was relatively low and similar to IL4, as found in our experimental results. Naïve T cell-initiated B cell proliferation stimulated the prompt increase of mucus IgA, IgE and IgG above the activation threshold (**Fig. 5C**). The consequent recruitment of neutrophils, along with IgG, led to the reduction but not clearance of adult helminths, consistent with the empirical observation that a few individuals still harboured helminths in the duodenum at 120 days post infection (**Fig. 5A**). Unlike IgA, whose activity followed the dynamics of *T. retortaeformis* abundance, IgG activity remained persistently high. In contrast to the small and short-lived neutrophil peak, the eosinophil activity was higher and lasted longer (**Fig. 5D**).

The stability of the immune pathways and the reliability of our parsimonious model were explored by systematically knocking out network nodes and examining the effects on the activity of the adult helminth node at the 20^th^ time-step, the time point when the unperturbed system reaches equilibrium (**Fig. 4C**). None of the perturbations led to an activity of the adult parasite node of less than 0.3, indicating that *T. retortaeformis* persists in the rabbit and this is a robust outcome of the model, which matches our empirical observations. Simulations suggested that the individual knockout of 14 nodes, including pro-inflammatory cytokines, IL13, naïve T cells, dendritic cells, eosinophils and neutrophils led to helminth persistence in all the simulations (i.e. adult activity equal to 1) (**Fig. 4C**). Interestingly, deletion of either local or systemic IL4 (IL4I or IL4II) reduced parasite activity, as IL4 contributed to inhibit neutrophils (via the inhibition of the IL12 node). To identify the nodes that may lead to faster reduction or clearance of *T. retortaeformis* we constitutively turned ON single nodes. Over-expression of recruited eosinophils, IL5, neutrophils and Th2 cells in the small intestine reduced parasite activity below 0.5 (results not shown). These and the knockout simulations suggested that neutrophils and eosinophils are critically involved in the clearance of *T. retortaeformis* infection.

### *B. bronchiseptica-T. retortaeformis* co-infection

#### Network modelling

**Figure 6:**
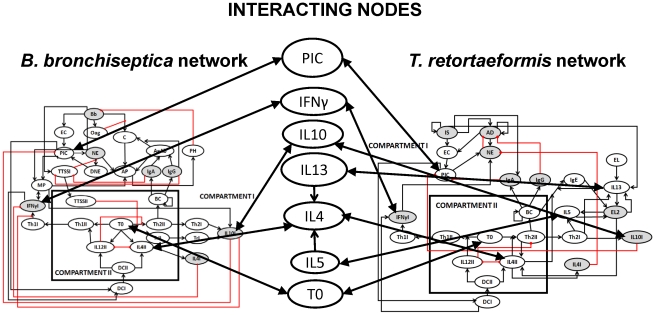
Network of immune components considered in the *B. bronchiseptica-T. retortaeformis* co-infection. Bi-directional black arrows indicate the influence of components from one network on the common cytokine pool and vice a versa.

To explicitly quantify the interactions between *B. bronchiseptica* and *T. retortaeformis* the two single immune networks were connected and the co-infection network simulated as a single entity without changing the Boolean rules built for the single networks, except for the adjustments necessary for assembly (**Fig. 6**). The link between networks was established through the cytokines, which maintain the communication between the systemic and local immune processes as well as the cross-interactions between infections. Specifically, we assumed a single unlimited pool of naive T cells and three pools of cytokines: a pool in the lungs, a pool in the small intestine (duodenum) and a systemic pool interacting with both infections. For example, we assumed that only one pool of IL4 and IL12 exists in the systemic compartment although antigen specific cells, polarized towards bacteria or helminths, can produce these cytokines. In other words, IL12 induced by bacterial factors can inhibit IL4 production by helminth-specific Th2 cells. Local cytokine expression can be affected by mucosal immune components, parasite intensity and the systemic cytokine response. These assumptions allowed us to take into account the compartmentalization of the infections (i.e. lungs and duodenum) as well as bystander effects of the immune response and the balance of the immune system as a whole. The dynamics of the simulated immune components and associated parasite activity were then compared with the empirical co-infection results.

#### *B. bronchiseptica*

**Figure 7:**
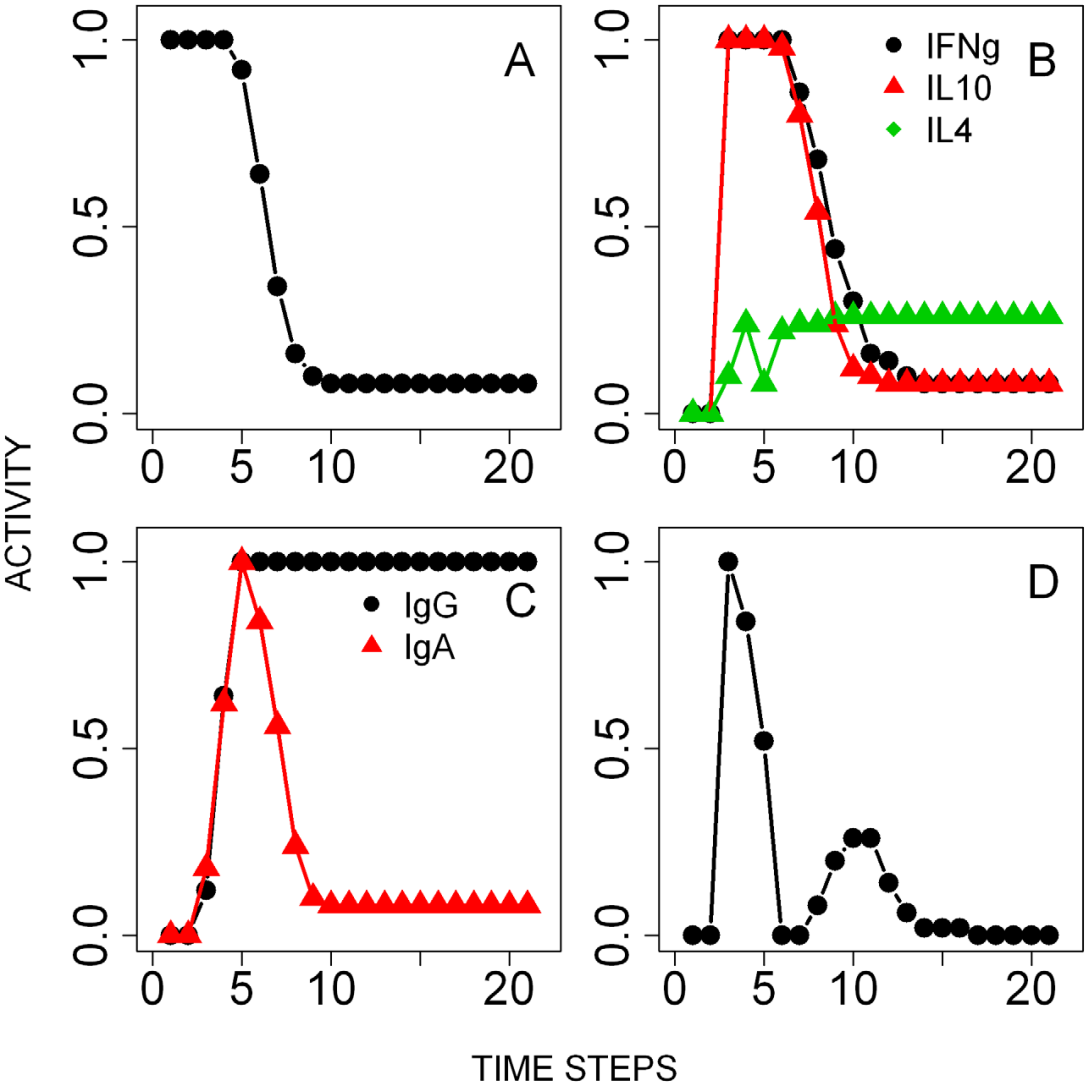
Results of the simulations of the time course of *B. bronchiseptica* from the co-infection. Activity profiles (the probability of the node being in an ON state at a given time-step) are reported for: **A**- Bacterial colonies in the lungs. **B**- Cytokines, IFNγ, IL4 and IL10, in the lungs. **C**- Serum antibodies. **D**- Peripheral neutrophils.

Simulations showed the switch of cytokines from the initial high expression of IFNγ and IL10 to the late increase and long activity of IL4 (**Fig. 7B**). Antibodies quickly increased, serum IgG remained consistently high while IgA decreased below the threshold after 5 time-steps as bacterial numbers declined (**Fig. 7C**). The peripheral neutrophil activity was higher in co-infected compared to single infected hosts, however, their recruitment in the lungs was completely turned off after 14 time steps (**Fig. 7D** *vs* **Fig. 5D**). These temporal patterns resulted from the inflammatory cytokines produced in response to both *T. retortaeformis* and *B. bronchiseptica* and should be interpreted as a mixed activity against both parasites. Our simulations indicated similarities between *B. bronchiseptica* single and co-infection, such as the rapid increase in systemic IgA, IgG and neutrophils but also differences, namely, the higher and longer activity of IL4 in the lungs and the longer presence of peripheral neutrophils in dual compared to single infection. Overall, despite a few immunological differences the dynamics and timing of *B. bronchiseptica* clearance in the lungs of co-infected hosts was similar to that observed in the single infection and driven by phagocytic cells activated by antibodies and Th1 cells (**Fig. 7A**). The low but non-zero activity of bacteria in the co-infection steady state indicated that the infection was not cleared in a small fraction of the replicate simulations (8%) (**Fig. 7A**). Specifically, IL4 activated by eosinophils in response to *T. retortaeformis* was responsible for the persistence of bacteria in the lungs. During single bacterial infection the IL4 level was relatively low and controlled by the inhibitory effect of IL12, however, during the co-infection this suppressive effect was not observed as a Th2 environment dominated. This model prediction is supported by previous studies that showed a delayed bacterial clearance in case of persistent IL4 [42]. Knockout perturbation analysis confirmed that IL4 produced by eosinophils was responsible for this occasional bacterial persistence, since the deletion of this node led to the complete clearance of the infection in all the simulations (**Fig. 4B**). Bacterial persistence was also observed when Th1 cells, antibodies, pro-inflammatory cytokines or the activated phagocytes node were individually knocked out. The 15 nodes whose deletion had very little effect in the single infection had a similarly weak effect on bacterial numbers in the co-infection (**Fig. 4A** *vs* **4B**). Interestingly and contrary to the single infection, the knockout of bacteria-activated epithelial cells did not influence *B. bronchiseptica* activity since the pro-inflammatory cytokines node, which is downstream of the epithelial cells node, was also activated by the helminths. This between-organ communication was possible by assuming a single pool of cytokines and their free movement among organs, for example via the blood system. Perturbation of any of the 17 helminth-specific nodes had a generally weak effect on bacterial activity.

#### *T. retortaeformis*

**Figure 8:**
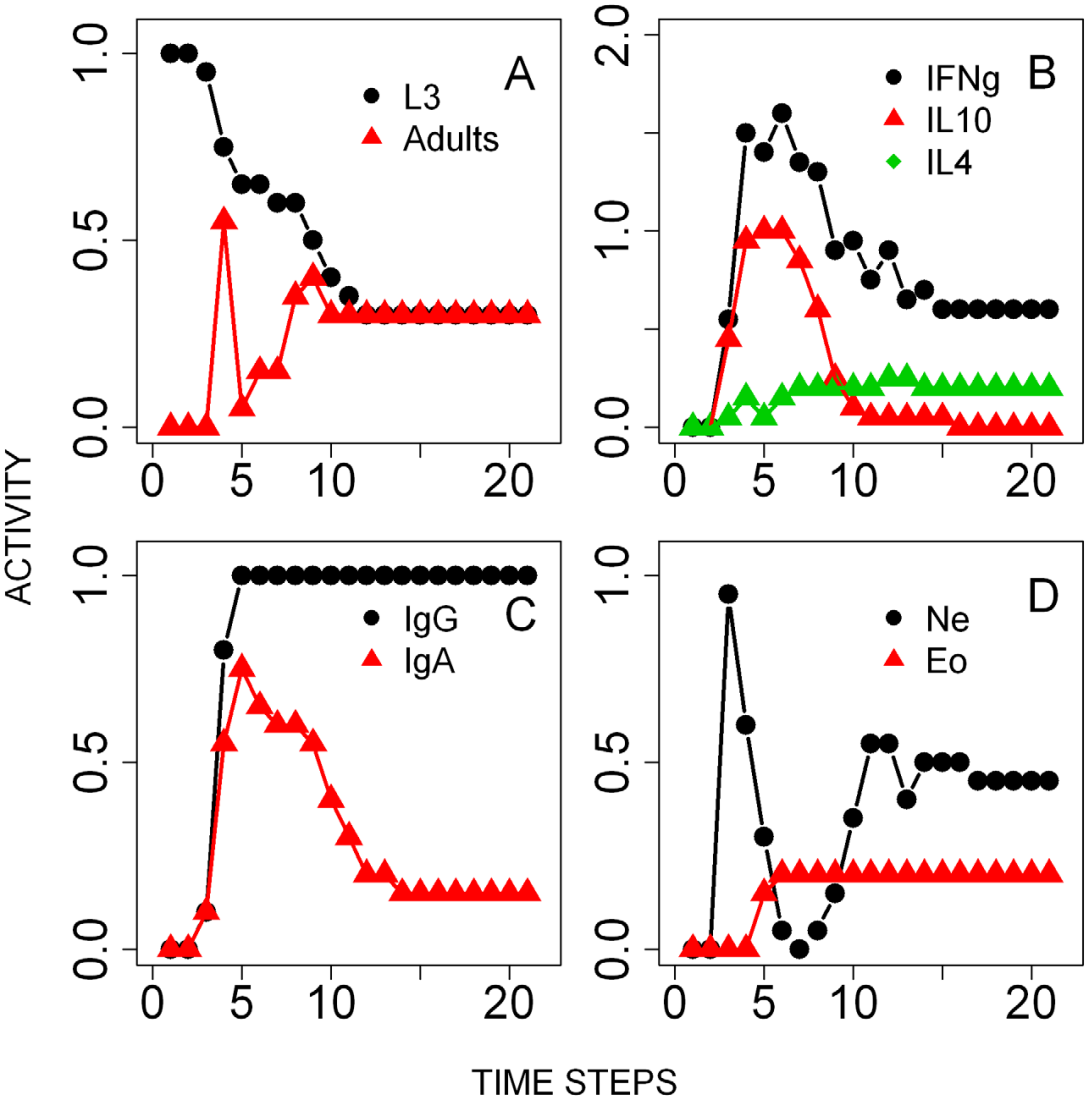
Results of the simulations of the time course of *T. retortaeformis* infection from the co-infection. Activity profiles (the probability of the node being in an ON state at a given time-step) are reported for: **A**- Third stage infective larvae (L3) and adults. **B**- Cytokines, IFNγ, IL4 and IL10 in the duodenum. **C**- Mucus antibodies against adult helminths. **D**- Peripheral eosinophils and neutrophils. Note that the IFNγ concentration range is between 0–2 to describe additional non-immune mediated activation of that node by the tissue damage (details in the Results).

The concurrent effect of *B. bronchiseptica* on *T. retortaeformis* infection dynamics was equally examined. Counter to our initial predictions, lower establishment and faster clearance of *T. retortaeformis* were observed in co-infected compared to single infected hosts (**Fig. 8A** *vs* **5A**). The model showed high activities of IFNγ and IL10 and low expression of IL4 (**Fig. 8B**). As observed in the single infection, the early peak of IFNγ (having activity >1) was caused by an initial host-mediated inflammatory response, as an immediate-type hypersensitivity reaction of the tissue to the establishment of infective larvae. This local activation was then followed by a Th1 mediated IFNγ expression, consistent with the single infection model. A bystander Th1 mediated effect of *B. bronchiseptica* synergistically contributed to this pattern by enhancing the activity and duration of IFNγ expression in the duodenum. Simulations suggested that the local IL10 expression, higher in the dual compared to the single infection, was a bystander effect induced by the type three secretion system (TTSS) of *B. bronchiseptica* through Treg cells. Also, the early IL4 expression was suppressed by the Th1 mediated IFNγ phenotype activated both by the helminth, during the initial establishment, and the bacterial co-infection. Mucus IgG remained consistently active from time step 3 while mucus IgA was at the highest between 5 and 10 time steps but decreased thereafter (**Fig. 8C**). Recruited peripheral neutrophils but not eosinophils were higher in the dual infection compared to single helminth infection simulations (**Fig. 8D**).

To provide a parsimonious mechanism that could explain the rapid helminth clearance, the immune nodes of the co-infection network were systematically knocked out and the helminth activity examined at the 20^th^ time-step (**Fig. 4D**). Similar to the single infection, the deactivation of key nodes, for instance B cells, dendritic cells or T cells, resulted in helminth persistence in all the simulations (adult activity equal to 1). Unlike in the single infection, knockout of resident eosinophils or the IL12II node did not lead to helminth persistence. This was because the induction of downstream processes, such as the activation of IL4 or IFNγ was now performed through the complementary effect of the bacterial nodes and their bystander effects. Interestingly, the single knockout of 92% of the nodes, including bacterium-specific nodes, increased helminth activity, compared to the unperturbed co-infection model, but did not lead to helminth persistence in every simulation. From a modelling perspective, the network in Fig. 6 represents a sparse causal model of co-infection dynamics. In other words, all these nodes or nodes downstream of the targeted nodes contribute to, but are not required for, *T. retortaeformis* clearance. The knockout of effector nodes namely, recruited eosinophils or neutrophils and cytokines like IL5 or IL13, resulted in helminth long term persistence, supporting the hypothesis that a co-operative mechanism including leukocytes, antigen-specific antibodies (IgG and IgE) and Th2 mediated IL5 and IL13 are critical in helminth clearance [43]–[49]. The role of IL5 and IL13 is mostly in the recruitment of eosinophils while neutrophils are recruited by pro-inflammatory cytokines and Th1 mediated IFNγ. Though antibodies recognize the helminth, in this model they do not form complexes, rather, they attract leukocytes bearing Fc-receptors leading to the recruitment of neutrophils and eosinophils.

A comparison between single and dual infection offers insights into the contribution and balance of these two leukocytes to *T. retortaeformis* dynamics. In the single infection, when neutrophils are only transiently activated, the recruited eosinophils were relatively more important to parasite reduction, although they were not sufficient to clear the infection. In the co-infection, the robust and early activation of recruited neutrophils -which decreased following helminth reduction- and the activation of recruited eosinophils -which are important in reducing the number of infecting larvae and are required for neutrophils to successfully reduce the helminths- highlighted the synergistic role of these cells in the observed fast clearance of *T. retortaeformis*. To explicitly study the bacterial components inducing these two leukocytes, we switched the nodes to ON one at a time and found that dendritic cells and Th1 cells, activated by bacteria, led to a significant increase in neutrophil activity (results not shown). Counter to this, no bacterial nodes significantly contributed to eosinophil production. Switching ON the type III secretion system node transiently increased eosinophil activity, compared to the unperturbed system, as expected from the role of TTSS in the induction of Th2 related cytokines [25]. However, this had a very short lived effect since TTSS was neutralized by antibodies. In summary, simulations suggest that strong inflammatory responses generated by the bacteria led to an early increase of neutrophils which contributed to a prompt and more effective helminth reduction.

### Empirical co-infection experiment

A *B. bronchiseptica-T. retortaeformis* co-infection experiment was carried out and the empirical results were used to validate the co-infection dynamic model. A statistical analysis was also performed between the single and co-infection trials to further reinforce our modelling outputs. However, while the statistical findings provide an insight into the relationships among the immune components, no mechanistic understanding or dynamic outcomes can be established between these variables and parasite abundance. The network-based discrete dynamic models allowed us to establish such connections and causal interactions between the various components. Overall, we found that the parsimonious dynamic model correctly predicted the observed dynamics of concurrent *B. bronchiseptica* and *T. retortaeformis* co-infection.

#### *B. bronchiseptica*

**Figure 9:**
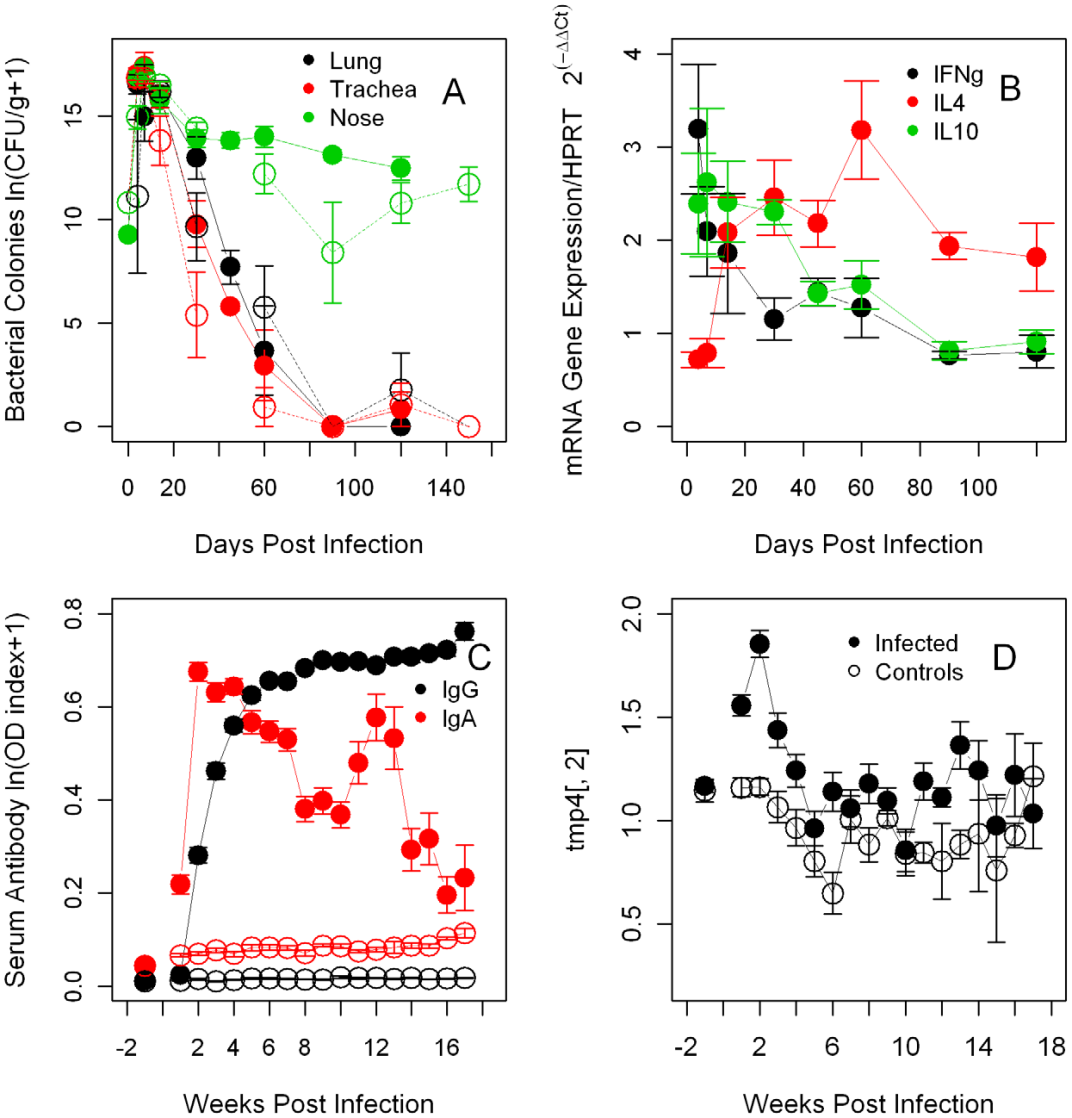
Summary of *B. bronchiseptica* intensity and immune variables from the experimental co-infection. Mean±SE during the course of the infection (days or weeks post infection) are reported. **A**- Bacterial intensity in the respiratory tract. For comparison, empty black circles represent the bacterial intensity in the lungs from the single infection. **B**- Cytokines, IFNγ, IL4 and IL10 in the lungs. **C**- Anti-bacterial IgA and IgG in serum. **D**- Peripheral neutrophils. For C and D, infected hosts: full circles, controls: empty circles.

**Figure 10:**
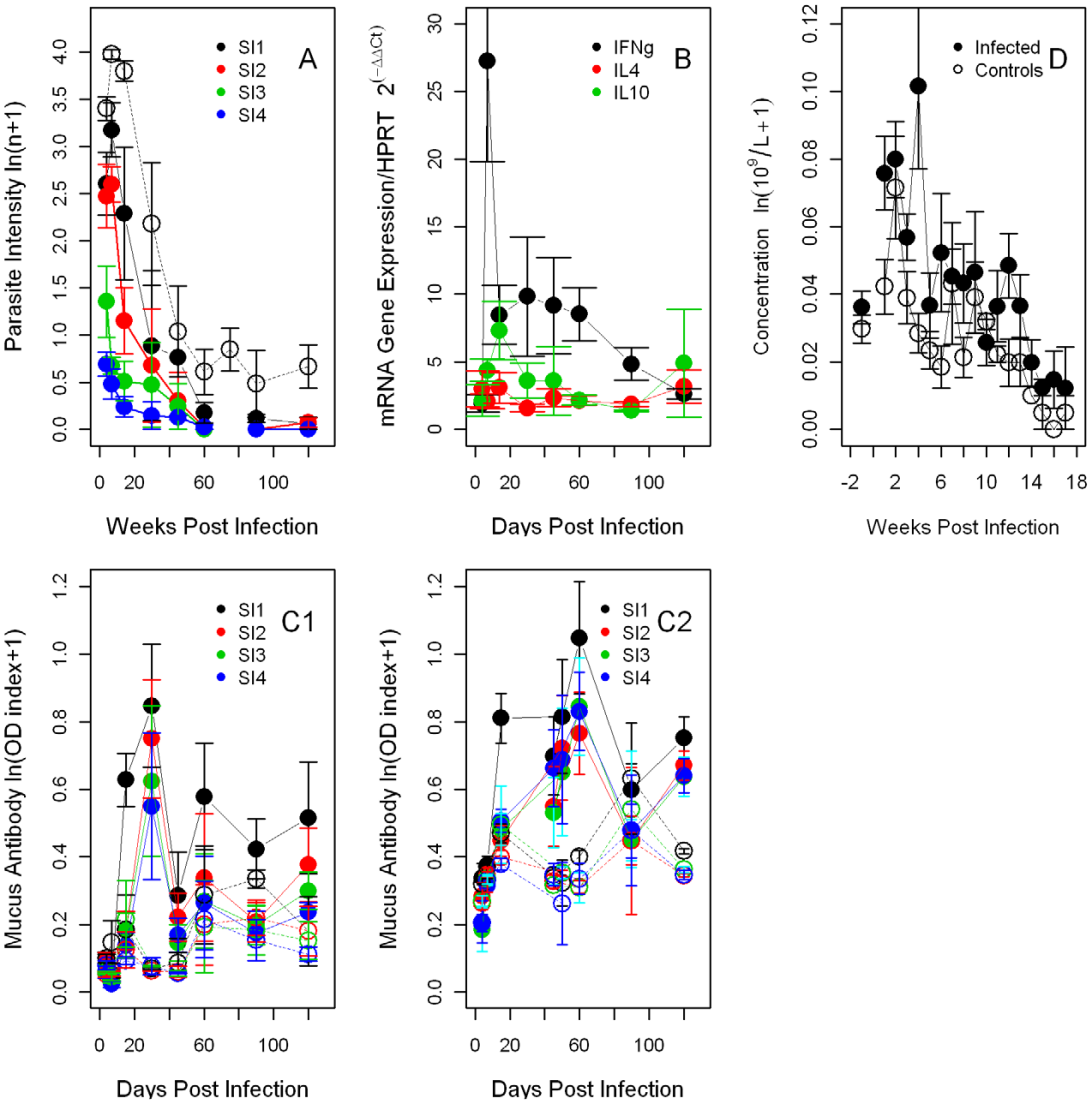
Summary of *T. retortaeformis* intensity and immune variables from the experimental co-infection. Mean±SE during the course of the infection (days or weeks post infection) are reported. **A**- Helminth intensity in the small intestine sections, from the duodenum (SI-1) to the ileum (SI-4), respectively. The helminth development during the course of the infection is as follows: 4 days post infection (DPI) third stage infective larvae (L3), 7 DPI both L3 and fourth stage larvae (L4), from 14 DPI onwards adult stage only. For comparison, empty black circles represent the helminth intensity in the duodenum from the single infection. **B**- Expression of cytokines, IFNγ, IL4 and IL10 in the duodenum. **C**- Mucus antibody against adult helminths, IgA (C1) and IgG (C2), from the duodenum to the ileum. **D**- Peripheral eosinophils. For C and D, infected hosts: full circles, controls: empty circles.

The bacterial colonization of the respiratory tract of co-infected rabbits was similar to single infection. *B. bronchiseptica* abundance in the lungs increased in the first 7 days post challenge and decreased thereafter, as seen in the dynamic model; by 90 days bacteria were completely cleared from the lungs and trachea but persisted in the nasal cavity (**Fig. 9A**). Based on the *a priori* measurement of optical density with a spectrophotometer, individuals received a dose similar to the single infection however, the *a posteriori* quantification of bacteria on blood agar plates suggested that an inoculum of 10,600 CFU/ml was administered, five times less than the single infection dose [28]. If we consider the second measure correct, the lower dose did not affect replication and the colony numbers quickly reached values comparable to single infection by 3–7 days post challenge. Specifically, the average number of bacteria in the lower respiratory tract was analogous to the single infection but significantly higher numbers were observed in the nasal cavity during the infection (**Fig. 9A** **,** **Table 1**). Confirming the model simulations, IFNγ quickly increased, peaked by 3 days post challenge and quickly decreased thereafter. IL10 followed a similar pattern with a small delay while IL4 slowly increased and peaked 60 days post infection (**Fig. 9B**). Serum antibodies showed a trend similar to that of the single infection, in accordance with our dynamic model. IgG rapidly increased and remained high throughout the experiment while IgA rapidly decreased although a second peak was observed around week twelve, this second peak was based on much fewer individuals and, probably, it was not biologically relevant (**Fig. 9C**). Peripheral leukocytes concentration reflected the response to both infections specifically, neutrophil numbers showed a robust peak at week three while eosinophil numbers increased between two and five weeks post-infection, both in agreement with the model (**Fig. 9D** and **Fig. 10D**).

**Table 1:** Summary of linear mixed effect model (LME) between *B. bronchiseptica* abundance (CFU/g), as a response, and infection type (single or co-infection), day post infection (DPI) and organ (lung, trachea or nose) as independent variables.

	Coeff±S.E., d.f.	P
Intercept	14.483±0.745, 122	0.00001
Infection type	1.711±0.895, 60	0.061
Trachea	−0.259±0.661, 122	0.695
Nose	0.721±0.757, 122	0.343
DPI	−0.113±0.010, 60	0.00001
Infection type*DPI	−0.045±0.015, 60	0.005
Trachea*DPI	−0.008±0.010, 122	0.425
Nose*DPI	0.076±0.011, 122	0.00001
Infection type*Trachea*DPI	0.004±0.013, 122	0.745
Infection type*Nose*DPI	0.039±0.015, 122	0.009
AIC	1022.895	
Host ID random effect (intercept S.D.)	1.113	
AR(1)	0.311	

The random effect of the host identity code (ID) and the autocorrelation effect (AR-1) of sampling different organs for the same host are also reported.

A combination of principal component analysis (PCA) and generalized linear models (GLM) indicated that *B. bronchiseptica* in the lungs was negatively associated with IL4, serum IgG and IgA (PCA axis 1), and peripheral eosinophils and neutrophils (PCA 2, **Table S1**). To compare the immune response between single and co-infected hosts, variables were scaled over the controls. Co-infected rabbits exhibited higher IL4 (coeff±S.E. = −0.879±0.210, P<0.001), serum IgG (0.166±0.043 P<0.001) and neutrophils (0.233±0.050, P<0.0001) but lower eosinophils (−1.705±0.006, P<0.0001) compared to single infected individuals. It is important to note that a low or negative cytokine Ct value (cycle threshold scaled over the controls) identifies high mRNA expression and vice versa, thus in the models low Ct values are translated as high cytokine activity. The remaining variables were not significant, although this should not be interpreted as a complete lack of variability between the two infections. Indeed, as highlighted in the network model these variables play a secondary but still necessary role in generating immune differences between infections.

#### *T. retortaeformis*

Helminth intensity significantly decreased with the progression of the infection and organ location (high numbers in the duodenum, SI1, and low in the ileum, SI4) however, counter to our expectation and consistent with our model simulations, lower establishment and faster clearance were observed in co-infected compared to single infected hosts (**Fig. 10A** **,** **Table 2**). As predicted by our dynamic model, strong and persistent IFNγ expression but relatively low IL4 and IL10 were found in the duodenum of infected rabbits compared to the controls (**Fig. 10B**). Consistent with the single infection and the dynamic model, mucus antibody quickly increased, IgG remained relatively high for the duration of the trial while IgA declined from day 30 post challenge (**Fig. 10C**). The peripheral leukocyte profile has already been described in the bacteria section (**Fig. 9D** and **Fig. 10D**). Principal component analysis identified that *T. retortaeformis* was positively associated with the first axis (PCA 1), mainly described by the interaction among the three cytokines, and negatively related to the second axis (PCA 2) represented by eosinophils and antibodies (**Table S2**). Interestingly, cytokines were positively correlated (IFNγ vs IL10: r = 58% P<0.001; IL4 vs IL10: r = 54%, P<0.01), indicating the co-occurrence of a specific response to the helminth, through IL4, but also a robust inflammatory/anti-inflammatory reaction probably caused by the parasite damaging the mucosal epithelium and resulting in bacterial tissue infiltration during larval establishment [31]. The comparison of immune variables between single and co-infection showed higher neutrophils (P<0.0001) and a tendency for higher IL10 (P = 0.058) in co-infected compared to single infected hosts. The overall expression of IL4 was lower in co-infected individuals (P = 0.035), however higher values were observed at 14 days post infection (interaction of IL4 with day 14 post infection P = 0.046).

**Table 2:** Summary of linear mixed effect model (LME) between *T. retortaeformis* abundance (worm/small intestine length) as a response, and infection type (single or co-infection), day post infection (DPI) and organ location (from the duodenum -SI1- to the ileum -SI4-), as independent variables.

	Coeff±S.E., d.f.	P
Intercept	3.044±0.178, 207	0.00001
Infection type	−0.778±0.240, 68	0.002
SI-2	−0.526±0.103, 207	0.001
SI-3	−1.766±0.138, 207	0.00001
SI-4	−2.530±0.159, 207	0.00001
DPI	−0.029±0.003, 68	0.00001
Infection type*SI-2	0.045±0.109, 207	0.682
Infection type*SI-3	0.243±0.146, 207	0.097
Infection type*SI-4	0.520±0.169, 207	0.001
Infection type*DPI	0.005±0.004, 68	0.214
SI-2*DPI	0.003±0.001, 207	0.023
SI-3*DPI	0.016±0.002, 207	0.00001
SI-4*DPI	0.023±0.002, 207	0.00001
AIC	500.453	
Host ID random effect (intercept S.D.)	0.001	
AR(1)	0.773	

The random effect of the host identity code (ID) and the autocorrelation effect (AR-1) of sampling different organs of the same host are also reported.

## Discussion

Co-infections affect the immune responses but how the systemic processes interact and influence the kinetics at the local sites of infection is still unclear. The majority of studies on the immunology of co-infection have focused on either one of the infecting species or a restricted class of cells or immune processes, and often concentrated on the early stage of the infection [4], [9]–[14], [50]–[52]. These studies have been extremely useful in highlighting not only the similarities across systems but also the specificity of some of these mechanisms and how they differ from single infections. Yet, there is a need for a comprehensive understanding of these processes as a whole individual response, how systemic and localized processes interact and how they dynamically evolve during the course of the co-infection. We used a combination of laboratory experiments and modelling to examine the dynamic network of immune responses to the respiratory bacterium *B. bronchiseptica* and the gastrointestinal helminth *T. retortaeformis*. Our aim was to identify the parsimonious processes and key cells driving parasite reduction or clearance and how they changed between single and co-infections.

We confirmed the initial hypothesis of immune mediated interactions between the two parasites, however, our initial predictions were only partially supported. The most unexpected result was the faster clearance of *T. retortaeformis* in co-infected compared to single infected individuals, which was observed in the model simulations and confirmed in the empirical data. Neither did we expect to find that *B. bronchiseptica* infection in the lungs was not significantly altered by the concurrent helminth infection, despite the increase in local IL4 expression observed in both the simulations and the experiment. We found a small difference in bacterial clearance between single and co-infection (**Fig. 3A** *vs* **Fig. 7A**) and we were able to explain that this was driven by the differential recruitment of phagocytes, particularly macrophages induced by IFNγ during co-infections, as compared to the single infection. However we found that *T. retortaeformis* enhanced individual variability in the immune response to *B. bronchiseptica* infection by occasionally reducing the overall efficacy of the Th1 immune response, through eosinophil produced IL4, and preventing bacterial clearance in the lungs, a pattern observed in 8% of the simulations. The helminth mediated delay or absence of bacterial clearance from the lower respiratory tract was indeed our original hypothesis and interestingly the model indicated that this is still a possible outcome of the interaction between these parasites. This implies that heterogeneities in the host immune response are not exceptional events and can have major effects on the dynamics of infection and persistence. Our model was able to capture this variability because of the large number of simulations; in other words a large group of infected individuals were examined compared to our much smaller sample tested in the laboratory. Follow-up experiments using a much larger number of animals or replication of the same experiment a few times may lead to the experimental observation of this behaviour. The empirical findings also showed that *T. retortaeformis* infection resulted in a significant increase of bacterial numbers in the nasal cavity compared to single infection, particularly after the initial phase of the infection. At the host population level these findings support the hypothesis that co-infections can increase individual variability to infections by altering bacterial intensity and prevalence, and this can have major consequences for the risk of transmission and disease outbreak [53]. Overall, our dynamic models indicated that the clearance of *B. bronchiseptica* in single and co-infection was mainly driven by phagocytosis of bacteria by macrophages and neutrophils activated by antibodies. Deactivating nodes that affected bacterial recognition (e.g. pro-inflammatory cytokines, epithelial cells or antibodies) or phagocytosis (e.g. Ag-Ab complex or macrophages) increased bacterial abundance in single and dual infections, suggesting that these cells are necessary for controlling *B. bronchiseptica*.

The immune network for *T. retortaeformis* was less detailed than that for the bacterial network, nevertheless, the model predictions of the activity pattern of the helminth and the immune variables that have been quantified were in agreement with our empirical studies. To our surprise the prediction of no effect of *B. bronchiseptica* on *T. retortaeformis* infection was proven wrong. Simulations suggested that the combined effect of neutrophils, eosinophils and antibodies (IgG and IgE) led to helminth expulsion. Neutrophils and eosinophils were activated through antigen-specific Th1 and Th2 responses, respectively. Th2-mediated differentiation of progenitor eosinophils (i.e. resident eosinophils), modulated by IL5 and IL13, also played an important role in helminth reduction in single infection, as indicated by the perturbation results. Previous studies on murine systems have shown that IL13 can complement IL4 or play an alternative or even stronger role in helminth infections [42]–[43]. Using our modelling approach we showed that IL5 and IL13 had complementary abilities against helminths and contributed to parasite reduction both in single and co-infection. The strategic role of neutrophils in bacteria-helminth co-infections has been previously described [44]; using a modelling approach not only we confirmed this property but also suggested a non-specific infiltration of effector cells into infected tissues.

The mixed Th1/Th2 response in the duodenum was driven by different processes. The early IFNγ inflammatory signal observed both in single and co-infection was a host response to the mucosa damage by helminth establishment, and probably bacteria and microflora infiltration from the lumen [31]. This was also complemented by a bystander effect of *B. bronchiseptica* co-infection, rather than a helminth induced up-regulation of this cytokine to facilitate tissue colonization [31]. This mechanism is supported by our recent studies on cytokine expression in different organs of single and co-infected rabbits at seven days post infection, where we showed that IFNγ was remarkably reduced in the ileum, mesenteric lymph node and spleen, where fewer or no helminths were found, compared to the duodenum [54]. The Th2 cell activity was primarily focused on preventing parasite establishment and survival. These findings indicate that these two cytokines are not mutually exclusive but can simultaneously act on different tasks specifically, tissue repair, inflammatory response to microflora infiltration and helminth clearance. Mixed Th1/Th2 phenotypes are not new to parasite infections and the murine-*Schistosoma mansoni* or *Trichuris muris* systems are well described examples [55]–[57].

### Model strengths and limitations

The aim of this study was to develop tractable dynamic models that could capture the interactions of multi-organ, multi-species co-infection immune processes as well as single infection dynamics. We found the discrete dynamic Boolean models a feasible and reliable approach for this task since we lacked accurate spatio-temporal details on the majority of the variables and the kinetic parameters required to develop robust quantitative, differential equation-based models [34]–[36]. Boolean models assume that what matters the most is whether the concentration or level of expression of a node (i.e. immune cell) is higher or lower than an *a priori* fixed threshold. They also use a parameter-free combinatorial description for the change in status of the nodes, thus avoid the need for parameter estimation while being sufficiently flexible. Indeed, Boolean models have been successfully used in a variety of contexts, from signal transduction [38], [58] to development [59]–[60], immune responses [8], [61]–[62] and population-level networks [63]. Choosing a quantitative modelling approach would have forced us to drastically simplify our system, impose a large number of assumptions on the concentration, transfer function and kinetic parameter of each node, and so we would have not been able to offer robust predictions on the role of many immune components and on how they affect the dynamics of parasite infection in our system.

Our models were based on the most updated knowledge of the immune components and processes during single infections to *Bordetella* and gastrointestinal helminths. In cases of uncertainty (e.g. whether two co-regulators were independent or synergistic) we tested a number of different assumptions (i.e. Boolean transfer functions) and selected the function that best described our single infection experiments in terms of the: timing of events, node activities and importantly, parasite steady state (see Materials and Methods for an example). To overcome the fact that timescales and duration of immune processes were unknown, we generated repeated simulations with various update orders, which essentially allowed us the sampling of various time durations and probing which model output was robust to timing uncertainties. Importantly, the outputs of our simulations were not averages but the quantification of the agreement between runs, for example, the anti-*B. bronchiseptica* IgG activity of 1 after step 4 in Fig. 3C means that following this time point all runs show an above-threshold concentration of IgG regardless of timing variations. By comparing the features of the curves (e.g. saturating shape, peak occurrence and timing) with our experimental observations we were able to confirm the accuracy of the model in predicting the observed kinetics.

One of the strengths of our modelling was to make predictions on the dynamics of parasite clearance based on the perturbation of the nodes (i.e. single node knockout). These simulations followed the classical knockout lab experiments where single immune components (nodes) were turned off from the beginning of the simulation and the dynamics of the immune response, as well as parasite clearance, were examined. This approach allowed us to explore the knockout of a large number of immune variables, determine the most important components modulating the immune response and highlight how they differed between single and co-infection. These findings can be tested in the laboratory by performing knockout experiments of the crucial immune variables in different infection settings. For example, we can block neutrophil production or the cytokine IL13 and examine whether helminths persist -as predicted by our knockout simulations- or are slowly cleared in bacteria co-infected rabbits. Similarly, we can test the predicted different response of knocking out IL4 in helminth and bacteria-helminth co-infection, specifically, whether clearance is higher than in un-manipulated individuals in single helminth infection and lower than in un-manipulated co-infected hosts. We should also pay more attention to *B. bronchiseptica* infection in the nasal cavity and develop dynamic immune models that can explain bacterial persistence as well as possible clearance under different knockout scenarios both in single and co-infection. The most parsimonious hypotheses can then be tested in the laboratory. This is important because our recent work suggested that bacterial shedding during the long lasting chronic phase relies mainly on the infection of the upper respiratory tract, once it has been cleared from the lungs and trachea [28]. This has relevant epidemiological implications for bacterial transmission that go beyond the rabbit-parasite system. We can further refine our models and explore the dynamics of the parasite-immune network when the onset of the co-infections is lagged between the parasite species or one parasite is trickle dosed, a dynamic that resembles more closely to the natural conditions. Again, these predictions can be validated through experimental infections of naïve or knockout animals. It is important to underline that our approach can be adapted to a large variety of bacteria-helminth co-infections of many host systems where organ compartmentalization, differences in the time of infection or number of parasite stages are observed.

In conclusion, we showed that network-based discrete dynamic models are a useful approach to describe the immune mediated dynamics of co-infections. These models are robust as well as sufficiently tractable to qualitatively capture the complexity of the immune system and its kinetics over time. Arguably, the main limitation of our modelling approach is that it lacks a fully quantitative component. Yet, this work demonstrated that it is possible to build comprehensive qualitative dynamic models of the local and systemic immune network of single and co-infection that are validated by empirical observations. Importantly, this study is a fundamental starting point towards the future construction of quantitative models based on simplified networks that describe the kinetics and intensities of the causal relationships among key immune components identified in qualitative models. Our approach showed that we can refine the conventional approach of using the Th1/Th2 paradigm, by identifying system-specific functions or cell groups that can capture crucial immune processes during co-infections. While our parsimonious dynamical models were able to capture the patterns of single and co-infection observed in the experiments, we are aware that they are far from complete in describing the immunological complexity of the processes involved and cells activated. Nevertheless, they provide a parsimonious description of the system that can be experimentally tested. Ultimately, we showed that we cannot predict how the immune system reacts to co-infections based on our knowledge of single infection. More needs to be done to clarify the immune mechanisms involved in bacteria-helminth co-infections and how individual hosts balance the immune system as a whole.

## Materials and Methods

### Network modelling

#### Network assembly

Interaction networks were built from the available literature and adapted to our system. Bacteria, helminth and the components of the immune system (i.e. immune cells and cytokines) were represented as network nodes; interactions, regulatory relationships and transformations among components were described as directed edges starting from the source node (regulator) and ending in the target node. We incorporated regulatory relationships that modulate a process (or an unspecified process mediator) as edges directed toward another edge. The regulatory effect of each edge was classified into activation or inhibition, visually represented by an incoming black arrow or an incoming red blunt segment. Since not all processes involved in natural *B. bronchiseptica* and *T. retortaeformis* infections are known or generally addressed in the rabbit infection model, we extended the set of known interactions following general immunological knowledge on bacterial and helminth infections. We constructed three networks: two networks that describe the respective single infections and one that links the first two and represents a co-infection network. A detailed description of each network is given below.

#### *B. bronchiseptica* single infection

Infection of the lungs starts with the node Bacteria that leads to a cascade of immune interactions (**Fig. 1** **, Text S1**). This node includes generic virulence factors of the bacteria such as the lipopolysaccharide chain (LPS) required for tissue adherence following recognition of bacteria by epithelial cells. Other bacterial virulence factors, particularly O-antigen and type III secretion system (TTSS), are explicitly included as separate nodes in the network and are involved in the initial immune recognition of the bacteria node. Upon detection, epithelial cells activate pro-inflammatory cytokines, which in turn activate dendritic cells, often the most important antigen presenting cells. Dendritic cells are also activated by IFNγ. Dendritic cells induce differentiation of naïve T cells (T0) by producing IL4 and IL12. The cytokine profile along with the antigen leads to the activation of T cell subtypes including helper and regulatory T cells. T helper cells are activated in the lymph nodes (Compartment II) and subsequently transported to the site of infection (Compartment I). IL4 is also produced by differentiated Th2 cells; IL4 and IL12 inhibit each other and IL4 also inhibits IFNγ. T regulatory (Treg) cells are stimulated by the type III secretion system of *B. bronchiseptica* to produce IL10. Th1 cells produce IFNγ which along with pro-inflammatory cytokines activates neutrophils and macrophages. A different subtype of T cells, follicular T helper cells, is known to stimulate B cell activation. To simplify the network we assumed that naïve T cells could play this role. Antigen-specific B cell proliferation leads to the production of antibodies, namely IgG and IgA. IgA production occurs only in the direct presence of antigen unlike IgG that persists after bacterial clearance [28]. IgG and bacteria complexes also induce complement fixation along with bacteria themselves. Activation of complement by bacteria is inhibited by O-antigen. The node “activated phagocytic cells” represents the outcome of the stimulation of neutrophils and macrophages by antibody-antigen complex and complement. These cells induce the node phagocytosis that depletes bacteria.

#### *T. retortaeformis* single infection

The network starts with infective larvae that develop into adults with no delay in the larval-adult development, adults appear 2 time steps post infection (**Fig. 2** **, Text S1**). Both parasite stages activate epithelial cells that lead to the production of pro-inflammatory cytokines which then activate dendritic cells and neutrophils, with the latter able to inhibit adult helminths. Infective larvae stimulate IL13 production by resident eosinophils and these recruit additional eosinophils from the progenitor cells in the peripheral blood [63]. Eosinophils can kill larvae through a stochastic process described by a uniform distribution [64]. IL5 secreted by Th2 cells is required for the recruitment of additional eosinophils. Infective larvae also directly activate IFNγ by damaging the mucosa tissue and causing a host inflammatory response. This process does not include Th1 cells. Pro-inflammatory cytokines activate dendritic cells that stimulate naïve T cells (T0). As described for *B. bronchiseptica*, dendritic cells interact with naïve T cells (T0) leading to the activation of T cell subtypes Th1 and Th2 through the production of IL12 and IL4. IL4 is also produced by Th2 cells and IL4 and IL12 inhibit each other. Consistent with the bacteria network, the activation of T helper cells occurs in the lymph nodes (Compartment II) and subsequently transported to the site of infection (Compartment I). In compartment I, IFNγ is produced by Th1 cells and dendritic cells. IL4 and IL10, produced by Th2 cells, have anti-inflammatory properties and inhibit pro-inflammatory cytokines and neutrophils. Naive T cells stimulate clonal expansion of B cells and these lead to the production of antibodies such as IgG. While B cells can secrete IgG much longer after antigen removal, IgA production is assumed to be in response to larval establishment and development. The IgE isotype is produced upon signalling from either IL4 or IL13. Among these antibodies IgG inhibits adult helminths while IgE and IgA are involved in activating eosinophils and inhibiting pro-inflammatory cytokines respectively.

#### *B. bronchiseptica-T. retortaeformis* co-infection

The co-infection immune network was developed by combining the two single infection networks together (**Fig. 6** **, Text S1**). This network is characterized by three compartments, representing the lungs, the small intestine (duodenum) and the systemic compartment (e.g. the lymphatic system). The connection of the networks and the immune mediated interactions between parasites were represented through the cytokines produced as a single pool. Local cells activated by bacteria and helminths can contribute to cytokine production, which are then transported through the blood and disseminate to other organs [63]. For example, pro-inflammatory cytokines are systematically detectable when any one of the parasites activates epithelial cells. Similarly, IL4 or IL12 can be produced by *B. bronchiseptica*- or *T. retortaeformis*-specific T subtypes or dendritic cells. For the co-infection network, Tregs are induced by bacteria which produce IL10 that can ultimately affect the helminth, since IL10 is not an antigen-specific node. Moreover, there is only a single pool of naïve T cells that induces T cell subtypes against either the bacteria or the helminths, depending on the antigen-specific dendritic cells.

#### Discrete dynamic model implementation

The immune-parasite interaction networks were developed into discrete dynamical models by characterizing each node with a variable that can take the ON state, when the concentration or activity is above the threshold level necessary to activate downstream immune processes, or the OFF state when activity is below this threshold. The evolution of the state of each node was described by a Boolean transfer function (**Text S1**) [32]. Target nodes with a single activator and no inhibitors follow the state of the activator with a delay. The operator AND was used to describe a synergistic or conditional interaction between two or more nodes that is necessary to activate the target node. When either of the nodes were sufficient for the activation of the target node we used the operator OR. An inhibitory effect was represented by an AND NOT operator. In cases where prior biological information did not completely determine the transfer functions (e.g. there was no information whether two coincident regulatory effects are independent or synergistic), different alternative transfer functions were tested. The transfer functions that reproduced the qualitative features of the single infection experimental time courses, such as the parasite clearance profile, the relative peaks of different cytokines or the saturating behaviour of IgG as compared to IgA, were selected. For example, IL4 is produced by T helper cells during T helper cell differentiation as well as by eosinophils in response to stimulation by nematode antigens or allergens. While IL12 is known to inhibit the production of IL4, there are two possible ways this cytokine may interact with IL4: IL12 can inhibit IL4 produced by T helper cells or IL12 can suppress IL4 production by blocking both the T helper and eosinophil signal. The inhibitory effect of IL4 on the activation of neutrophils is known. The two transfer functions were then examined by comparing the temporal pattern for neutrophils and IL4 from the single *T. retortaeformis* infection model with the experimental observations. The second transfer function did not reproduce the observed low activity of IL4 -compared to the other cytokines- in the duodenum at day 14 post infection and it also led to higher neutrophil activity, compared to the other leukocytes, than the empirical data. Since the first transfer function did not lead to such deficiencies, we chose the first over the second rule. The transfer functions used in the co-infection model were the same as, or the relevant composites of, transfer functions used in each individual infection. Thus, the Boolean transfer functions applied in our model provide a mechanistic understanding of the interactions leading to bacterial or helminth clearance.

The status of the system across time was simulated by repeatedly applying the Boolean rules for each node until a stationary state (e.g. clearance of the parasite) was found. Since the kinetics and timescales of the individual processes represented as edges are not known, a random order asynchronous update was selected wherein the timescales of each regulatory process were randomly chosen in such a way that the node states were updated in a randomly selected order during each time-step [32]. The asynchronous algorithm was: 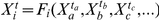, where *F* is the Boolean transfer function, *t_a_, t_b_, t_c_* represent the time points corresponding to the last change in the state of the input nodes *a, b, c* and can be in the previous or current time-step. The time-step (time unit) of our model approximately corresponds to nine days. The randomized asynchronicity of the model does not alter the steady states of the dynamical system but causes stochasticity in the trajectory between the initial conditions and the equilibria (attractors) [32], [37], thus it can sample more diverse behaviours as the traditionally used synchronous models. To determine the node consensus activity over time (i.e. shared by trajectories with different update orders) we ran the simulations 100 times and presented the fraction of simulations in which the node was in an ON state at a given time-step in the node activity profile. We confirmed that running the simulations for more than 100 times did not change the activity profiles.

Our approach of using discrete dynamic modelling allowed us to sample the timescales of interactions and perform replicate simulations as well as provide continuously varying activities of the network nodes over time, which ranged between the lower limit of 0 (below-threshold concentration in all runs) and upper limit of 1 (above-threshold concentration in all runs). However, notice the exception for IFNγ expression higher than one in the helminth infections. While these activities cannot be directly compared to quantitative concentrations, we could compare the qualitative features of the time courses and ask: are they saturating? Do they show single or multiple peaks? We could also compare the relative trends of similar variables. It is important to stress that the empirical data on *B. bronchiseptica-T. retortaeformis* co-infection were not used as inputs to the co-infection model but only to validate the simulated course and intensity of immune responses during co-infection.

### Laboratory experiments

The primary single infections of naïve rabbits with *B. bronchiseptica* strain RB50 and *T. retortaeformis* have been described in detail in Pathak et al. [28] and Murphy et al. [31]. The co-infection of naïve rabbits with a primary dose of *B. bronchiseptica* RB50 and *T. retortaeformis* followed similar procedures. Here, we report a concise description of the experimental design, quantification of the immune variables and parasite intensities.

#### Ethics statement

All listed animal procedures were pre-approved by the Institutional Animal Care and Use Committee of The Pennsylvania State University.

#### Co-infection study design

Out-bred 60 days old New Zealand White male rabbits were intra-nasally inoculated with 1 ml of PBS solution containing 2.5×10^4^ *B. bronchiseptica* RB50 and simultaneously orally challenged with a 5 ml mineral water solution of 5,500 infective third stage *T. retortaeformis* larvae (L3). Control individuals were treated with 1 ml of PBS or 5 ml of water, respectively. Groups of 6 individuals (4 infected and 2 controls) were euthanized at days 3, 7, 14, 30, 60, 90, 120 post challenge and both the respiratory tract and small intestine were removed to quantify: parasite abundance, cytokine expression in the lungs and small intestine (duodenum) and mucus-specific anti-helminth antibody levels (IgA and IgG) from the duodenum to the ileum (Section SI-1 to SI-4). Blood samples were collected weekly and used for serum-specific antibody quantification against both parasites and leukocyte cells count [28], [31].

#### Parasite quantification

A fixed amount of lungs (15 ml), trachea (5 ml) and nasal cavity (15 ml), homogenized in PBS, was serial diluted onto BG blood agar plates supplemented with streptomycin and incubated at 37°C for 48 hours for bacteria quantification (Colony forming units, CFU) [28]. The four sections of the small intestine (SI-1 to SI-4) were washed over a sieve (100 µm) and helminths collected and stored in 50 ml tubes. Parasites were counted in five 2.5 ml aliquots and the mean number, developmental stage and sex (only for adults) estimated in the four sections [31].

#### Local cytokine gene expression

The expression of IFNγ, IL-4 and IL-10 in the lung and duodenum was determined using Taqman qRT- PCR. RNA isolation, reverse transcription and qRT-PCR quantification were performed following protocols we have developed [28], [31].

*Antibody detection:* Antibody IgA and IgG against *B. bronchiseptica* and adult *T. retortaeformis* were quantified using Enzyme-Linked Immunosorbance Assay (ELISA) [28], [31]. Optimal dilutions and detector antibody against the two parasites were selected by visually identifying the inflection point from the resulting dilution curves. For *B. bronchiseptica* serum dilutions were: 1∶10 for IgA and 1∶10,000 for IgG, secondary detection antibody: IgA 1∶5,000 and IgG 1∶10,000. For *T. retortaeformis* mucus dilution was: 1∶10 both for IgA and IgG and 1∶5,000 for the secondary antibody. We found cross-reactivity at the antibody level between the somatic third stage infective larvae (L3) and the adults both in the serum and the mucus [31]. As such and for simplicity, the empirical data and the network models were based on the antibody response to the adult helminth stage.

#### Haematology

Blood in anti-coagulated EDTA tubes was processed using the Hemavet 3 haematology system (Drew Scientific, USA) and the general haematological profile quantified [28].

#### Statistical analysis

Linear mixed effect models (LME-REML) were applied to identify changes in the immune variables during the course of the co-infection and between single and co-infection. The individual identification code (ID) was included as a random effect and an autoregressive function of order 1 (AR-1) was integrated to take into account the non-independent sampling of the same individual through time or the monitoring of different parts of the same organ from the same individual. To identify the combination of immunological variables that mainly affected parasite abundance a principal component analysis (PCA singular value decomposition) was used [31]. Briefly, the strongest linear combination of variables along the two main PC axes was identified; generalized linear models (GLM) were then used to examine how parasite abundance was influenced by each PC axis. To compare the immune variables between single and co-infection, data from infected animals were initially scaled over the controls as: Xij* = Xij-Xc, where *Xij* is an immune variable for individual *i* at time *j* and *Xc* is the total average of the controls across the infection for that variable.

## Supporting Information

Table S1 Relationship between *B. bronchiseptica* abundance (CFU/g) and immune variables from the co-infection experiment. **A-** Summary of the Principal Component Analysis (PCA) based on the most representative immune variables; only the first two PCA axes are reported. Note that the cytokine Ct values are inversely related to the level of expression. **B-** Summary of the generalized linear model (GLM) between bacteria abundance and PCA axis 1 and axis 2.
(DOC)

Table S2 Relationship between *T. retortaeformis* abundance (worm/duodenum length) and immune variables from the co-infection experiment. **A-** Summary of the Principal Component Analysis (PCA) based on the most representative immune variables; only the first two PCA axes are reported. Note that the cytokine Ct values are inversely related to the level of expression. **B-** Summary of the generalized linear model (GLM) between helminth abundance and PCA axis 1 and axis 2.
(DOC)

Text S1 Transfer functions for every node of each network: **A-** Single *B. bronchiseptica* infection; **B-** single *T. retortaeformis* infection; **C-** *B. bronchiseptica-T. retortaeformis* co-infection. In the functions we depict the nodes in the intestine with the suffix ‘t’ and the nodes in the lungs with the suffix ‘b’. Abbreviations: **Oag**: O-antigen; **IL4II**: Interleukin 4 in systemic compartment; **DNE**: Dead neutrophils; **NE**: Recruited neutrophils; **IL12I**: Interleukin 12 in lungs/intestine; **IgA**: Antibody A; **C**: Complement; **TrII**: T regulatory cells in systemic compartment; **IL4I**: Interleukin 4 in lungs/small intestine; **Th2II**: Th2 cells in systemic compartment; **TrI**: T regulatory cells in lungs/small intestine; **Th2I**: Th2 cells in lungs/small intestine; **IL10II**: Interleukin 10 in systemic compartment; **TTSSII**: Type three secretion system in systemic compartment; **TTSSI**: Type three secretion system in lungs; **IgG**: Antibody G; **IgE**: Antibody E; **IL10I**: Interleukin 10 in lungs/small intestine; **IFNγII**: Interferon gamma in systemic compartment; **IFNγI**: Interferon gamma in lungs/small intestine; **IL12II**: Interleukin 12 in systemic compartment; **BC**: B cells; **DCII**: Dendritic cells in systemic compartment; **DCI**: Dendritic cells in lungs/small intestine; **Th1I**: T helper cells subtype I in lungs/small intestine; **PIC**: Pro-inflammatory cytokines; **Th1II**: T helper cells subtype I in systemic compartment **EC**: Epithelial cells lungs/intestine; **AP**: Activated phagocytes; **T0**: Naïve T cells; **AgAb**: Antigen-antibody complexes; **MP**: Macrophages in lungs; **EL2**: recruited eosinophils; **EL**: resident eosinophils; **IL13**: Interleukin 13; **IL5**: Interleukin 5; **TEL**: total eosinophils; **TNE**: total neutrophils; **TR**: *T. retortaeformis*, **Bb**: *B. bronchiseptica* **DNE**: dead neutrophils; **IS**: *T. retortaeformis* Larvae; **AD**: *T. retortaeformis* Adults; **PH**: Phagocytosis.
(DOC)
