# Supplementary material for: Network Model of Immune Responses Reveals Key Effectors to Single and Co-infection Dynamics by a Respiratory Bacterium and a Gastrointestinal Helminth
Source: PLoS Comput Biol. 2012 Jan 12;8(1):e1002345. doi: 10.1371/journal.pcbi.1002345 (PMC3257297; doi:10.1371/journal.pcbi.1002345)
Supplement: Table S1 — Relationship between B. bronchiseptica abundance (CFU/g) and immune variables from the co-infection experiment. A- Summary of the Principal Component Analysis (PCA) based on the most representative immune variables; only the first two PCA axes are reported. Note that the cytokine Ct values are inversely related to the level of expression. B- Summary of the generalized linear model (GLM) between bacteria abundance and PCA axis 1 and axis 2. (DOC) [file pcbi.1002345.s001.doc]

**THAKAR ET AL. SUPPORTING INFORMATION**

**Table S1.** Relationship between *B. bronchiseptica* abundance (CFU/g) and immune variables from the co-infection experiment. **A-** Summary of the Principal Component Analysis (PCA) based on the most representative immune variables; only the first two PCA axes are reported. Note that the cytokine Ct values are inversely related to the level of expression. **B-** Summary of the generalized linear model (GLM) between bacteria abundance and PCA axis 1 and axis 2.

| **(A)**  PCA | **PCA-1** | **PCA-2** |
| --- | --- | --- |
| IFNγ | -0.412 | 0.215 |
| IL4 | 0.479 | 0.132 |
| IL10 | -0.317 | -0.783 |
| IgA | -0.397 | -0.306 |
| IgG | -0.578 | 0.155 |
| Eosinophils | -0.089 | -0.646 |
| Neutrophils | 0.015 | -0.611 |
| St. Dev.; % Variance explained | 1.562; 34.9 | 1.307; 24.4 |
|  |  |  |
| **(B)** | **Coeff±S.E., d.f.** | **P** |
| Intercept | 0.147±0.0001 | 0.0001 |
| CFU vs PCA-1 | 0.866±0.0001, 29 | 0.0001 |
|  |  |  |
| Intercept | 0.155±0.0001 | <0.0001 |
| CFU vs PCA-2 | -0.443±0.0001, 29 | <0.0001 |
