# Supplementary material for: Network Model of Immune Responses Reveals Key Effectors to Single and Co-infection Dynamics by a Respiratory Bacterium and a Gastrointestinal Helminth
Source: PLoS Comput Biol. 2012 Jan 12;8(1):e1002345. doi: 10.1371/journal.pcbi.1002345 (PMC3257297; doi:10.1371/journal.pcbi.1002345)
Supplement: Table S2 — Relationship between T. retortaeformis abundance (worm/duodenum length) and immune variables from the co-infection experiment. A- Summary of the Principal Component Analysis (PCA) based on the most representative immune variables; only the first two PCA axes are reported. Note that the cytokine Ct values are inversely related to the level of expression. B- Summary of the generalized linear model (GLM) between helminth abundance and PCA axis 1 and axis 2. (DOC) [file pcbi.1002345.s002.doc]

**THAKAR ET AL. SUPPORTING INFORMATION**

**Table S2.** Relationship between *T. retortaeformis* abundance (worm/length duodenum) and immune variables from the co-infection experiment. **A-** Summary of the Principal Component Analysis (PCA) based on the most representative immune variables; only the first two PCA axes are reported. Note that the cytokine Ct values are inversely related to the level of expression. **B-** Summary of the generalized linear model (GLM) between helminth abundance and PCA axis 1 and axis 2.

| **(A)** PCA | **PCA-1** | **PCA-2** |
| --- | --- | --- |
| IFNγ | -0.443 | 0.297 |
| IL4 | -0.331 | 0.391 |
| IL10 | -0.518 | 0.365 |
| IgA | 0.426 | 0.443 |
| IgG | 0.345 | 0.378 |
| Eosinophils | 0.155 | 0.511 |
| Neutrophils | 0.318 | 0.161 |
| St. Dev.; % Variance explained | 1.501; 32.2 | 1.298; 24.1 |
|  |  |  |
| **(B)** | **Coeff±S.E., d.f.** | **P** |
| Intercept | 1.817±0.0745 | <0.0001 |
| Intensity vs PCA-1 | 0.265±0.048, 29 | <0.0001 |
|  |  |  |
| Intercept | 1.619±0.088 | <0.00001 |
| Intensity vs PCA-2 | -0.599±0.061, 29 | <0.0001 |
