# Supplementary material for: Network Model of Immune Responses Reveals Key Effectors to Single and Co-infection Dynamics by a Respiratory Bacterium and a Gastrointestinal Helminth
Source: PLoS Comput Biol. 2012 Jan 12;8(1):e1002345. doi: 10.1371/journal.pcbi.1002345 (PMC3257297; doi:10.1371/journal.pcbi.1002345)
Supplement: Text S1 — Transfer functions for every node of each network: A- Single B. bronchiseptica infection; B- single T. retortaeformis infection; C- B. bronchiseptica-T. retortaeformis co-infection. In the functions we depict the nodes in the intestine with the suffix ‘t’ and the nodes in the lungs with the suffix ‘b’. Abbreviations: Oag: O-antigen; IL4II: Interleukin 4 in systemic compartment; DNE: Dead neutrophils; NE: Recruited neutrophils; IL12I: Interleukin 12 in lungs/intestine; IgA: Antibody A; C: Complement; TrII: T regulatory cells in systemic compartment; IL4I: Interleukin 4 in lungs/small intestine; Th2II: Th2 cells in systemic compartment; TrI: T regulatory cells in lungs/small intestine; Th2I: Th2 cells in lungs/small intestine; IL10II: Interleukin 10 in systemic compartment; TTSSII: Type three secretion system in systemic compartment; TTSSI: Type three secretion system in lungs; IgG: Antibody G; IgE: Antibody E; IL10I: Interleukin 10 in lungs/small intestine; IFNγII: Interferon gamma in systemic compartment; IFNγI: Interferon gamma in lungs/small intestine; IL12II: Interleukin 12 in systemic compartment; BC: B cells; DCII: Dendritic cells in systemic compartment; DCI: Dendritic cells in lungs/small intestine; Th1I: T helper cells subtype I in lungs/small intestine; PIC: Pro-inflammatory cytokines; Th1II: T helper cells subtype I in systemic compartment EC: Epithelial cells lungs/intestine; AP: Activated phagocytes; T0: Naïve T cells; AgAb: Antigen-antibody complexes; MP: Macrophages in lungs; EL2: recruited eosinophils; EL: resident eosinophils; IL13: Interleukin 13; IL5: Interleukin 5; TEL: total eosinophils; TNE: total neutrophils; TR: T. retortaeformis, Bb: B. bronchiseptica DNE: dead neutrophils; IS: T. retortaeformis Larvae; AD: T. retortaeformis Adults; PH: Phagocytosis. (DOC) [file pcbi.1002345.s003.doc]

**THAKAR ET AL. SUPPORTING INFORMATION**

**Text S1.** Transfer functions for every node of each network: **A**- Single *B. bronchiseptica* infection; **B-** single *T. retortaeformis* infection; **C-** *B. bronchiseptica-T. retortaeformis* co-infection. * Represents future state in the current state *t+1*. In the functions we depict the nodes in the intestine with the suffix ‘t’ and the nodes in the lungs with the suffix ‘b’.

**Abbreviations: Oag**: O-antigen; **IL4II**: Interleukin 4 in systemic compartment; **NE**: Recruited neutrophils; **IL12I**: Interleukin 12 in lungs/intestine; **IgA**: Antibody A; **C**: Complement; **TrII**: T regulatory cells in systemic compartment; **IL4I**: Interleukin 4 in lungs/small intestine; **Th2II**: Th2 cells in systemic compartment; **TrI**: T regulatory cells in lungs/small intestine; **Th2I**: Th2 cells in lungs/small intestine; **IL10II**: Interleukin 10 in systemic compartment; **TTSSII**: Type three secretion system in systemic compartment; **TTSSI**: Type three secretion system in lungs; **IgG**: Antibody G; **IgE**: Antibody E; **IL10I**: Interleukin 10 in lungs/small intestine; **IFNγII**: Interferon gamma in systemic compartment; **IFNγI**: Interferon gamma in lungs/small intestine; **IL12II**: Interleukin 12 in systemic compartment; **BC**: B cells; **DCII**: Dendritic cells in systemic compartment; **DCI**: Dendritic cells in lungs/small intestine; **Th1I**: T helper cells subtype I in lungs/small intestine; **PIC**: Pro-inflammatory cytokines; **Th1II**: T helper cells subtype I in systemic compartment **EC**: Epithelial cells lungs/intestine; **AP**: Activated phagocytes; **T0**: Naïve T cells; **AgAb**: Antigen-antibody complexes; **MP**: Macrophages in lungs; **EL2**: recruited eosinophils; **EL**: resident eosinophils; **IL13**: Interleukin 13; **IL5**: Interleukin 5; **TEL**: total eosinophils; **TNE**: total neutrophils; **TR**: *T. retortaeformis*, **Bb**: *B. bronchiseptica* **DNE**: dead neutrophils; **IS**: *T. retortaeformis* Larvae; **AD**: *T. retortaeformis* Adults; **PH**: Phagocytosis.

**(A)**

Bb* = Bb and not PH

TTSSI* = Bb and not (IgG or IgA)

TTSSII* = TTSSI

Oag* = Bb

EC* = Bb

IgG* = BC or IgG

C* = (Bb and not Oag) or (AgAb and IgG)

AgAb* = Bb and (IgG or IgA)

IgA* = (BC or IgA) and Bb

BC* = T0 or BC

PIC* = (EC or DP or AP) and not IL10I

IL12I* = (DCII and T0 and not IL4II)

IL12II* = (DCII and T0 and not IL4II)

IL4I* = IL4II

IL4II* = ((DCII and T0) or Th2II) and not IL12II and not IFNγII

IL10I* = (MPI) or (Th2I and TTSSI) or TrI

IL10II* = IL10I

IFNγI* = ((MPI or DCI) or (Th1I and not IL10I)) and not IL4I

IFNγII* = IFNγI

NE* = PIC

DP* = NE and TTSSI

MPI* = (PIC or IFNγI) and Bb

AP* = Bb and (MPI and Th1I) and ((C and IgG) or AgAb)

T0* = DCII

TrII* = DCII and T0

TrI* = TrII

Th1II* = DCII and T0 and IL12II

Th1I* = Th1II

Th2II* = DCII and T0 and not IL12II

Th2I* = Th2II

DCI* = (IFNγI or PIC) and Bb

DCII* = DCI

PH* = AP and Bb

**(B)**

TEL* = EL or EL2

AD* = (IS or AD) and not (NE and IgG)

IS*=IS

IL13* = (IS and EL) or EL2 or Th2I

EL* = IS and not EL2

IgE* = BC and (IL4II or IL13)

IL5* = Th2I or EL2

EL2* = IL5 and (IgE or IL13)

EC* = IS or AD

IgG* = BC

IgA* = BC and IS

BC* = T0 or BC

PIC* = EC and not (IL10I or IgA)

IL12II* = DCII and T0 and not IL4II

IL4I* = IL4II

IL4II* = (((DCII and T0) or Th2II) ) and not (IL12II) or EL2

IL10I* = Th2I

IFNγI* = Th1I or DCI

NE* = (PIC and AD) or IFNγI and not (IL4I or IL10I)

T0* = DCII

Th1II* = DCII and T0 and IL12II

Th1I* = Th1II

Th2II* = DCII and T0 and not IL12II

Th2I* = Th2II

DCI* = PIC

DCII* = DCI

**(C) Note: bacteria specific nodes are with suffix “b” and helminth specific nodes are depicted with suffix “t”.**

TEL* = EL or EL2

AD* = (IS or AD) and not ((NEt or MPIb) and IgG)

IS*=IS

IL13* = (IS and EL) or EL2 or Th2It or Th2Ib

EL* = IS and not EL2

IgE* = BC and (IL4II or IL13)

IL5* = Th2It or EL2

EL2* = IL5 and (IgE or IL13)

ECt* = IS or AD

IgG* = BCt

IgA* = BCt and IS

BCt* = T0 or BCt

NEt* = (PIC and AD) or IFNγI and not (IL4I or IL10I)

T0* = DCIIt or DCIIb

Th1IIt* = DCIIt and T0 and IL12II

Th1It* = Th1IIt

Th2IIt* = DCIIt and T0 and not IL12II

Th2It* = Th2IIt

DCIt* = PIC

DCIIt* = DCIt

PIC* = (ECt or ECb or DP or AP) and not (IL10I or IgAt)

IL12II* = ((DCIIt and T0) or (DCIIb and T0)) and not IL4II

IL4I* = IL4II

IL4II* =((((DCIIt and T0) or (DCIIb and T0)) or (Th2IIt or Th2IIb) ) and not (IL12II or IFNγI)) or EL2

IL10I* = Th2It or IL10Ib

IFNγI* = DCIt or Th1It or IFNγIb

IFNγII* = IFNγI or IFNγIb

TNE*=NEt or NEb

Bb* = Bb and not PH

TTSSI* = Bb and not (IgGb or IgAb)

TTSSII* = TTSSI

Oag* = Bb

ECb* = Bb

IgGb* = BCb or IgGb

Cb* = (Bb and not Oag) or (AgAbb and IgGb)

AgAbb* = Bb and (IgGb or IgAb)

IgAb* = (BCb or IgAb) and Bb

BCb* = T0 or BCb

IL10Ib* = (MPIb) or (Th2Ib and TTSSI) or TrIb

IFNγIb* = (MPIb or DCIb) or (Th1Ib and not IL10Ib) and not IL4I

NEb* = PIC

DP* = NEb and TTSSI

MPIb* = (PIC or IFNγIb) and Bb

AP* = Bb and (MPIb and Th1Ib) and ((Cb and IgGb) or AgAbb)

TrII* = DCIIb and T0 and TTSSII

TrIb* = TrII

Th1IIb* = DCIIb and T0 and IL12II

Th1Ib* = Th1IIb

Th2IIb* = DCIIb and T0 and not IL12II

Th2Ib* = Th2IIb

DCIb* = (IFNγIb or PIC) and Bb

DCIIb* = DCIb

PH* = AP and Bb
